# Recognition of non-standard base pairs by triplex-forming oligonucleotides containing an expanded genetic alphabet

**DOI:** 10.1038/s41467-026-74375-4

**Published:** 2026-06-12

**Authors:** Michael Brazzill, Ruolin Ma, Kieron Munn, Léna Prestifilippo, Andrew R. Pickford, Hyo-Joong Kim, Cen Chen, Shuichi Hoshika, Steven A. Benner, David A. Rusling

**Affiliations:** 1 School of Medicine, Pharmacy and Biomedical Sciences, University of Portsmouth, Portsmouth, UK; 2 Polytech Angers, 16 Boulevard Daviers, Angers, France; 3 Centre for Enzyme Innovation, School of the Environment and Life Sciences, University of Portsmouth, Portsmouth, UK; 4 Foundation for Applied Molecular Evolution, Alachua, FL USA; 5 Firebird Biomolecular Sciences LLC, Alachua, FL USA

**Keywords:** Chemical biology, Biotechnology, Biophysics

## Abstract

The sequence-specific recognition of double-stranded DNA by biocompatible molecules is fundamental to molecular medicine and synthetic biology. Triplex-forming oligonucleotides (TFOs) enable programmable major groove recognition via Hoogsteen base pairing; however, the limited repertoire of natural nucleobases imposes strict constraints on target sequences and parallel motif triplexes require acidic conditions for stability. Here, we have expanded the triplex recognition space using nucleobases from an artificially expanded genetic information system (AEGIS). Through a systematic evaluation of 120 base triad combinations, we identify at least 12 modular triads that can be combined interchangeably to target duplex DNA containing standard, damaged, or synthetic base pairs with nanomolar affinity at neutral pH. We further demonstrate the versatility of this expanded recognition code by detecting oxidative lesions or AEGIS base pairs in enzymatically assembled duplex constructs using both chemically and enzymatically synthesized TFOs. This generalized framework provides a robust platform for precision gene-targeting, molecular sensing, and nucleic acid nanotechnology.

## Abstract (web summary)

Understanding the sequence-specific recognition of DNA by triplex-forming oligonucleotides (TFOs) is important for molecular medicine and synthetic biology. Here the authors use nucleobases from an artificially expanded genetic alphabet to systematically characterize 120 base triad combinations.

## Introduction

A central goal of molecular medicine and synthetic biology is to create biologically compatible molecules that can selectively bind unique double-stranded sequences in natural or synthetic DNA^1^. Such recognition agents should distinguish the Watson-Crick base pairs from the major or minor grooves based on their molecular shape, electrostatics, and/or hydrogen bonding capacity. Ideally, they should consist of modular recognition elements that can be strung together and used interchangeably to target sequences with varying base pair compositions and lengths. Moreover, their compatibility with enzymatic synthesis would not only simplify assembly but also enable their integration within biological systems, for example, as synthetic modulators of gene expression.

**Fig. 1:**
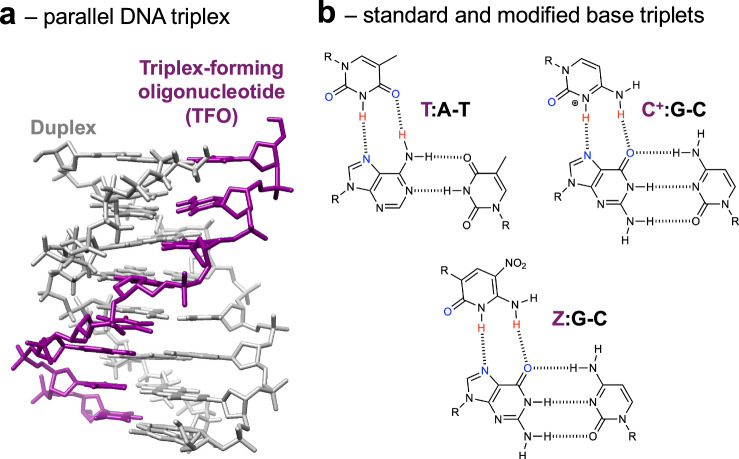
Triplex recognition by standard and modified nucleobases. **a** NMR structure of a parallel triplex in which a pyrimidine-rich TFO (purple) binds in the major groove of an oligopurine-oligopyrimidine duplex (gray) through the formation of specific base triad interactions (PDB code: 1D3X). **b** Chemical structure of **T**:A-T, **C**^**+**^:G-C, and **Z**:G-C triads formed through the interaction of the third strand base (X) with the Hoogsteen face of the purine (R) of a purine-pyrimidine (R-Y) base pair. The requirement for cytosine protonation limits triplex formation to acidic conditions, but can be alleviated using the AEGIS nucleobase Z, which mimics protonated cytosine and forms stable **Z**:G-C triads at neutral pH and above^19^.

Triplex-forming oligonucleotides (TFOs) are promising candidate molecules owing to their unique ability to bind specific DNA sequences in the major groove through programmable Hoogsteen base-pairing interactions, forming stable triplex structures (Fig. 1a)^2^. Synthetic TFOs have been used in vitro to detect and/or functionalize natural or synthetic DNA constructs^3–5^. They have shown potential as gene-targeting tools, modulating gene expression in vitro^6–8^, in cell culture, and in animal models^9,10^. Growing evidence also suggests that endogenous RNA modulates gene expression through triplex-based mechanisms^11^.

However, the standard nucleobases impose strict sequence, structural, and ionic constraints on triplex formation. Binding is typically limited to oligopurine-oligopyrimidine tracts, and pyrimidine-purine inversions are destabilizing. Purine-rich TFOs bind antiparallel to the purine strand of the duplex, forming **A**:A-T and **G**:G-C triads^12^. While pyrimidine-rich TFOs bind parallel, forming **T**:A-T and **C**^**+**^:G-C triads (Fig. 1b)^13,14^. [Here, we use X:R-Y to denote a triad, where the third strand base X interacts with the duplex base pair R-Y, by bonding to base R.] Parallel triplexes are inherently more stable than antiparallel triplexes because of the isostructural nature of their triads; however, they rely on cytosine protonation and therefore low pH ( < 6.0) conditions for assembly. Antiparallel oligonucleotides rich in purines can also readily form non-canonical secondary structures that compete with triplex formation^15,16^. For both motifs, stability relies on relatively high, yet physiologically relevant, cation concentrations to offset electrostatic repulsion between the three negatively charged strands^17^.

Much effort has focused on developing synthetic nucleotides to overcome the inherent limitations of triplex formation^2,18^. However, their incorporation into oligonucleotides often relies on bespoke phosphoramidite chemistry, which restricts their widespread use. An alternative strategy, pioneered by us and others, is to assemble TFOs from modified nucleotide triphosphates via templated or non-templated polymerase synthesis^19,20^. However, the structural complexity of existing nucleotides, often involving modifications to the base, sugar or phosphate, can hinder compatibility with polymerases. Nonetheless, such strategies have enabled gram-scale synthesis of modified oligonucleotides for antisense applications^21^.

**Fig. 2:**
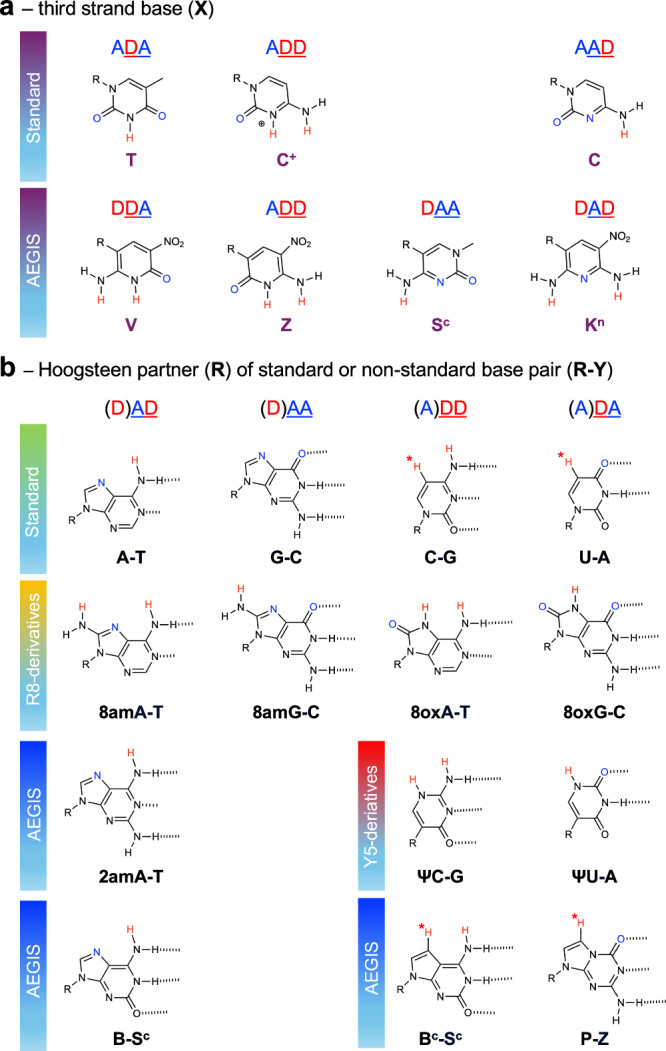
Chemical structures of standard and non-standard nucleobase and nucleobase pairs. **a** Standard and AEGIS “pyrimidines” collectively present nearly all possible hydrogen bond donor (D, red) and acceptor (A, blue) arrangements. **b** Their interactions were examined with base pairs involving standard bases, 8-substituted purines (R8) found in damaged DNA, 5-substituted pyrimidines (Y5), as well as AEGIS bases themselves. Pyrimidine bases bearing bulky 5-substituents (T, 5meC, 5ohU, 5ohC, and Z), along with the universal base analogue 5-nitroindole which exhibits reduced Hoogsteen bonding capacity, were also assessed but not expected to form stable triads (Supplementary Fig. 1). Recognition of purines typically involves hydrogen bond formation between the 3- and 4-positions of the third strand base and the 7- and 6-positions of the central purine (underlined). While recognition of pyrimidines generally involves bonding between the 2- and/or 3-positions of the third strand base and the 5- and 4-positions of the central pyrimidine. Non-conventional triad geometries are also possible (Supplementary Fig. 2). In all cases, unconventional C–H…O/N contacts may contribute to stability (indicated by red asterisks). The columns reflect the anticipated triad pairings based on hydrogen bond complementarity.

A potential route to overcome this bottleneck is to utilize nucleobases from an artificially expanded genetic information system (AEGIS)^22^. Their nucleotide triphosphates are compatible with natural or engineered polymerases, and the sequences can be amplified^23–28^, sequenced^29,30^, and even transcribed^31^. Moreover, their rearranged hydrogen bonding patterns create orthogonal duplex pairings distinct from those in natural DNA^32^ and could provide a new set of possible Hoogsteen triad pairing geometries. Perhaps the best-characterized of these duplexes pair the pyrimidines T, Z, S, K, and V (Fig. 2) with the purines D, P, B, X, and J, respectively. Four of these base pairs have been used alongside their natural counterparts to generate eight-letter “Hachimoji” DNA^31^. We have already established that the **Z** nucleobase is an effective mimic of protonated cytosine (Fig. 1b) and can be incorporated into TFOs via an adapted primer extension strategy^19^.

Here, we characterize the triplex-forming properties of the natural (**C** **T**) and all second-generation AEGIS pyrimidines (**Z** **S**^**c**^, **K**^**n**^, and **V**) within a pH-independent parallel triplex containing **Z**:G-C triads (Fig. 2a). Each nucleobase is assessed against 20 different base pair combinations presenting distinct Hoogsteen hydrogen bonding patterns in the major groove (Fig. 2b, Supplementary Fig. 1). These include the standard bases (A-T, G-C, C-G, T-A, 5meC-G, U-A), 8-derivatives of purines often found in damaged DNA (8amA-T, 8amG-C, 8oxA-T, 8oxG-C), 5-derivatives of pyrimidines (ΨU-A, ΨC-G, 5ohU-A, 5ohC-G), and AEGIS base pair themselves (2amA-T, B-S^c^, B^c^-S^c^, P-Z, Z-P). In doing so, we identify more than 12 non-standard base triads that, owing to their modular nature, can be used interchangeably to recognize DNA sequences containing multiple standard and synthetic base pair combinations with nanomolar affinity at neutral pH. We further highlight the practical utility of this new triplex recognition code by establishing that enzymatically assembled duplex constructs harboring oxidative lesions or AEGIS base pairs can be recognized by modified TFOs containing this expanded genetic alphabet.

## Results

### Triplex selectivity of an expanded genetic alphabet

**Fig. 3:**
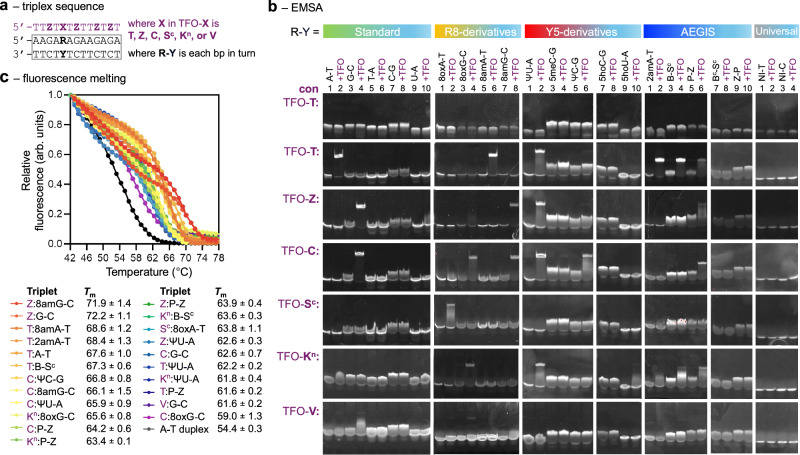
Triplex selectivity of an expanded genetic alphabet. **a** Triplex motif is used to assess the interaction of each nucleobase (**X**) with each base pair (R-Y) in turn. Oligonucleotides were annealed at pH 7.0 in a sodium cacodylate buffer containing 10 mM magnesium at a final duplex concentration of 1 μM and a TFO concentration of 2 μM. **b** Representative electrophoretic mobility shift assay for each sample (*n* = 2). Complexes were separated on a 20% non-denaturing polyacrylamide gel in standard tris-acetate running buffer and subjected to post-staining with GelRed. Triplex formation is evidenced by the reduced mobility of a sample relative to the duplex-only control. **c** Representative fluorescence melting profiles for the stable triads identified by EMSA screening (*n* = 3). Complexes were heated at 0.2 °C min^−1^ in the presence of SYBR Green I (Ex 488 nm/Em 522 nm). Separated melting profiles are shown in Supplementary Fig. 7. Melting temperatures (*T*_m_) indicate the mid-point of the melting transition calculated from first derivatives of the melting profiles using software provided by the machine, and the mean and standard error for each sample are shown. In each case, these were shifted to higher temperatures compared to the equivalent duplex-only samples (Supplementary Fig. 7). Thermal stability was dependent on the nature of the triad formed. Source data are provided as a Source Data file.

Here, we utilized a model triplex sequence from our previous work, in which a pyrimidine-rich 13-nucleotide TFO binds in a parallel orientation to a 13-base pair oligopurine-oligopyrimidine duplex containing four isolated G-C base pairs (Fig. 3a, Supplementary Table 2). Targeting of such a sequence using unmodified **CT**-containing TFOs is strongly pH dependent but alleviated by substituting cytosine with the AEGIS nucleobase **Z** (p*K*_a_ = 7.8)^19,33^. Such **ZT**-containing TFOs form highly stable complexes, generating triplexes that are more stable than the underlying duplex at a neutral pH^19,34^.

Using this pH-independent scaffold, we determined the interactions of the six pyrimidine nucleobases with 20 standard and non-standard base pairs, each presenting distinct Hoogsteen donor and acceptor arrangements in the major groove (Fig. 2, Supplementary Fig. 1). By systematically positioning each nucleobase opposite each base pair in turn, we evaluated 120 distinct **X**:R-Y triad combinations, where R and Y indicate bases positioned in the purine-rich and pyrimidine-rich strand of the duplex, respectively (Fig. 3a). Duplexes were first annealed at pH 7.0 in a sodium cacodylate buffer containing 10 mM magnesium and subsequently incubated with the appropriate TFO before examining the samples. Because most triplex applications operate in buffers containing 5–60 mM magnesium, we selected 10 mM as a useful reference point and as an approximation of physiological conditions.

Triplex formation was first assessed using an electrophoretic mobility shift assay (EMSA) at neutral pH (Fig. 3b). To maximize the sensitivity to differences in complex stability, electrophoresis was performed in a tris-acetate running buffer lacking magnesium^19,34^. Initial experiments with a **CT**-containing TFO (con TFO-**T**: Supplementary Table 2) confirmed that, as expected, the unmodified oligonucleotide did not bind to its duplex target at neutral pH (Fig. 3b). Only when the samples were prepared at pH 5.0 did the anticipated triplex—positioning thymine opposite an A-T base pair—form a stable complex, as evidenced by the reduced mobility of the sample (Supplementary Fig. 3). In contrast, all **ZT**-containing TFOs generated shifted species at neutral pH, with binding and stability influenced by the identity of the third strand base and the base pair positioned opposite.

Analysis of interactions with standard base pairs revealed that the behavior of the nucleobases largely reflected the different hydrogen bonding patterns presented in the major groove (Fig. 3b). A-T and G-C present donor-acceptor and acceptor-acceptor configurations, respectively; C-G and U-A present donor-donor and donor-acceptor arrangements when C–H…N/O interactions are considered^35^; whereas T-A contributes only a single acceptor together with a bulky methyl group that is known to destabilize triplex formation^36^. Consistent with these patterns, TFOs containing **T** **Z**, and **C** recognized A-T and G-C base pairs, forming established **T**:A-T, **Z**:G-C, and **C**:G-C base triads, respectively (*e.g*., Fig. 1a). Notably, the C:G-C triplet was tolerated in this stable sequence context, despite experiments being conducted at neutral pH. By contrast, TFOs containing **S**^**c**^ or **K**^**n**^ showed no detectable binding to any standard base pair, reflecting the incompatibility of their Hoogsteen pairing geometries.

An exception, however, was observed for the **V** nucleobase, which showed no interaction with the anticipated A-T base pair and instead displayed weak but reproducible binding to G-C. This was surprising because **V** mirrors the hydrogen bonding capacity of thymine at the 3- and 4-positions (Supplementary Fig. 4a). To test if selectivity was affected by sequence context, we investigated a second triplex motif positioning the base between **Z**:G-C triads, but again, the same preference for binding to G-C was observed by EMSA analysis (Supplementary Fig. 4b, Supplementary Table 3). One plausible explanation is that the presence of the 2-amino group, which is left uncompensated in a **V**:A-T triad, promotes the formation of a **V**:G-C triad by shifting the base further into the major groove (Supplementary Fig. 2c). The formation of such a non-conventional triad is expected to be destabilizing because it is not isostructural with **T**:A-T and **Z**:G-C triads (Supplementary Fig. 4a). Consistent with this interpretation, the incorporation of multiple **V**:G-C triads into the triplex sequence substantially reduced complex stability (Supplementary Fig. 4c).

Further analysis of the gels revealed that none of the nucleobases recognized any of the pyrimidine-purine inversions under the experimental conditions. This is consistent with the reduced donor/acceptor complementarity presented by pyrimidines and/or steric interference from bulky substituents at the 5-position of the pyrimidine ring. Experiments conducted at higher TFO concentrations and in the presence of additional magnesium revealed only modest recognition of a subset of these base pairs, confirming that such interactions are intrinsically disfavored (Supplementary Fig. 5b).

We next evaluated binding of the nucleobases to purines bearing 8-amino and 8-oxo substituents, which introduce additional hydrogen-bonding functionality in the major groove (Fig. 3b). The incorporation of an amino group at the 8-position of the purines was not expected to disrupt their recognition by **T,** **C**, or **Z** and could, in principle, form an additional hydrogen bond with the 2-keto group of the bases^37,38^. Consistent with this expectation, EMSA analysis revealed the formation of stable **T**:8amA-T, **C**:8amG-C, and **Z**:8amG-C triads. In contrast, the incorporation of a carbonyl substituent at the 8-position alters the Hoogsteen face of the purine, converting N7 from a hydrogen bond acceptor to a donor, thereby reversing the polarity of the Hoogsteen edge. Consequently, **T** and **Z** were not expected to recognize these modified purines, whereas **C** **S**^**c**^, and **K**^**n**^, which each present a potential acceptor at N3, were predicted to bind through acceptor–donor complementarity. Again, formation of these triads might also be stabilized by a third hydrogen bond between the 8-oxo group and the 2-amino group of the bases. Indeed, EMSA analysis confirmed the expected behavior, with **S**^**c**^:8oxA-T, **C**:8oxG-C, and **K**^**n**^:8oxG-C triads producing relatively stable complexes on the gels. However, some smearing and remaining duplex was observed for the triplex containing the **S**^**c**^:8oxA-T triad, indicating it was of lower stability compared to the other triads.

Similarly, we investigated whether non-natural pyrimidines bearing altered 5-position substituents influenced their triplex-forming capacity (Fig. 3b). The C-nucleosides ΨU and ΨC were of particular interest^39^, as both introduce an additional N-H donor into the major groove without perturbing their W-C pairings; although the recognition of ΨC will depend on its tautomeric equilibrium, which might be fixed upon duplex formation^40^. EMSA analysis revealed that **T** **Z** **C**, and **K**^**n**^ each formed detectable triads opposite a ΨU-A base pair, whereas only **C** could recognize ΨC-G. The formation of these triads is consistent with both conventional (**T**:ΨU-A, **Z**:ΨU-A, and **C**:ΨC-G) and non-conventional hydrogen bonding geometries (**C**:ΨU-A, and **K**^**n**^:ΨU-A) (Supplementary Fig. 2). We also assessed interactions with pyrimidines bearing bulky 5-position moieties and, irrespective of the potential to form hydrogen bonds, their presence strongly destabilized triplex formation; no interactions were observed with either 5-methyl (T and 5meC) or 5-hydroxy modified (5ohU and 5ohC) base pairs.

Finally, we assessed TFO binding to duplex sequences containing non-standard AEGIS base pairs (Fig. 3b). From the Hoogsteen face of the purine, both 2amA-T and B-S^c^ present the same acceptor-donor pattern as an A-T base pair; accordingly, thymine was expected to form stable **T**:2amA-T and **T**:B-S^c^ triads. EMSA analysis confirmed this prediction, revealing clear formation of both triads. In contrast, the P nucleobase displays a Hoogsteen edge not found in standard purines owing to the replacement of the N7 acceptor with a C-H moiety. Such a substituent might form a non-conventional C-H…O/N hydrogen bond with the nucleobases, for example, that is mediated by an N3 acceptor^35^. Interestingly, **T** **C** **Z**, and **K**^**n**^ all showed stable interactions with this base pair, reflecting the formation of both standard (**C**:P-Z and **K**^**n**^:P-Z) and non-standard (**T**:P-Z and **Z**:P-Z) triad geometries (Supplementary Fig. 2). We also assessed binding to the second-generation B^c^-S^c^ base pair, but owing to the removal of N7 and altered Hoogsteen face, triplex formation was not detected.

Additional control experiments confirmed that the formation of the modified triads requires hydrogen bonding, as none of the nucleobases interacted with the universal analogue 5-nitroindole, which lacks hydrogen bonding capacity (Fig. 3b). They also confirmed that the shifted species observed on the gels correspond to the same triplex architecture, as running the complexes side-by-side gave identical mobilities (Supplementary Fig. 5c). In addition, the selectivity of the nucleobases was not altered at either pH 5.0 or pH 9.0, despite the potential for protonation of **C** **S**^**c**^, and **K**^**n**^ at their N3 positions (Supplementary Fig. 6). The only exception was with **K**^**n**^, which exhibited a weak interaction with 8amG-C at low pH, suggesting that the nucleobase might be partially protonated at pH 5.0.

To quantify the thermal stability of the triads identified by EMSA analysis, we then performed fluorescence melting using SYBR Green I and a qPCR machine (Fig. 3c, Supplementary Fig. 7)^41^. SYBR green I fluoresces upon binding to duplex and triplex DNA^42^, and the melting of the complexes results in a decrease in fluorescence intensity at 522 nm^19^. Duplexes were first annealed at pH 7.0 in sodium cacodylate buffer containing 10 mM magnesium and subsequently incubated with the appropriate TFO before melting analysis. Triplex formation was identified by an upward shift in the melting profile relative to that of the corresponding duplex-only control. In some cases, we observed a minor drop in fluorescence at lower temperatures that can be attributed to the melting of unbound duplexes in the samples. Melting temperatures (*T*_m_) were therefore determined from the first derivatives of the highest melting transition and used to evaluate the relative stability of each triad. Due to its DNA binding mode, SYBR green I binding will lead to a slight stabilization of both the duplex and triplex complexes, and the *T*_m_ values determined will not equate to absolute *T*_m_ values obtained in the absence of the dye.

Examination of the melting profiles of the duplex sequences in the absence of TFO revealed modest differences in duplex stability, depending on the nature of the base pair under study (Supplementary Fig. 7a). As expected, the most stable base pairs (c*a*. 57 °C) were capable of forming three hydrogen bonds, except for B^c^-S^c^, which exhibited stability comparable to those containing two (*ca*. 53 °C). Melting profiles produced in the presence of all TFOs exhibited clear shifts in *T*_m_ values upon binding, with the extent of stabilization dictated by the identity of the base triad and distributed across a window of approximately 10 °C (Fig. 3c, Supplementary Fig. 7b). The relative stabilities of the most stable triads used later in the study followed the order **Z**:8amG-C = **Z**:G-C > **T**:8amA-T = **T**:2amA-T = **T**:A-T = **T**:B-S^c^ > **C**:ΨC-G > **K**^**n**^:8oxG-C > **C**:P-Z = **K**^**n**^:P-Z > **S**^**c**^:8oxA-T > **T**:ΨU-A = **T**:P-Z. All of these were predicted to form standard triad geometries except for T:P-Z, where the third strand T is shifted further into the major groove (Supplementary Fig. 2).

Surprisingly, under these conditions, **T**:8amA-T or **Z**:8amG-C triads were of a similar stability as their parent **T**:A-T and **Z**:G-C triads, respectively, despite the capacity to form three hydrogen bonds. The **K**^**n**^:8oxG-C and **S**^**c**^:8oxA-T triads also exhibited comparatively lower stability, despite the potential to form additional hydrogen bonding. However, previous work demonstrated that triplex stability is influenced by the stability of the underlying duplex and, consequently, thermal melting can underestimate stabilization afforded by nucleobase modifications^43^. Nevertheless, these experiments have established a new triplex recognition code that enables the rational targeting of duplex sequences containing standard, damaged, and non-standard base pairs.

### Modular recognition of different base pair combinations

**Fig. 4:**
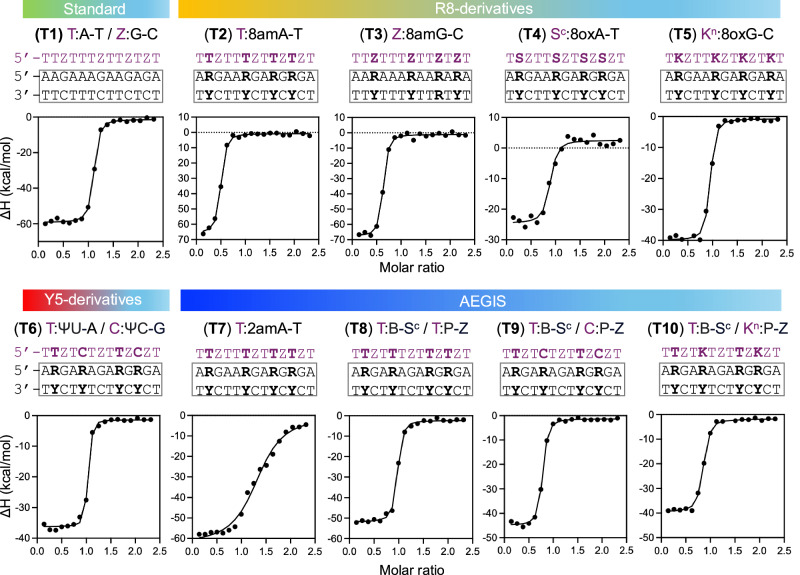
Modular recognition of different base pair combinations. Triplex motifs under study and varied in the type and number of modified base triads (T1-10). Oligonucleotides were dissolved in a pH 7.0 sodium cacodylate buffer containing 10 mM magnesium at a final duplex and TFO concentration of 5 μM and 60 μM, respectively. Each ITC experiment was conducted at 25 °C by titrating 19 aliquots of 4 uL TFO into the duplex sample with injections separated by intervals of 2.5 min. Triplex formation was evident for all duplex-TFO combinations, with each titration exhibiting an exothermic binding interaction and an affinity (*K*_D_) in the nanomolar range (Table 1). Source data are provided as a Source Data file.

To further assess the modularity and stability of this new triad recognition code, we designed a panel of oligonucleotide sequences to evaluate the formation of multiply modified triplexes comprised of the most stable isostructural triads identified above (Fig. 4). Starting from the parent construct comprising only **T**:A-T and **Z**:G-C triads (T1, Fig. 3a), nine additional sequences (T2-T10) were prepared (Supplementary Table 4). Constructs T2-T5 probed duplexes bearing multiple purine modifications via the formation of **T**:8amA-T, **Z**:8amG-C, **S**^**c**^:8oxA-T, and **K**^**n**^:8oxG-C triads, respectively. Construct T6 evaluated the recognition of a mixed sequence target containing ~33% pyrimidines through the incorporation of **T**:ΨU-A and **C**:ΨC-G triads. While sequences T7-T10 assess the recognition of duplexes harboring AEGIS base pairs, construct T7 evaluated the formation of multiple **T**:2amA-T triads, while constructs T8-T10 assessed recognition of Hachimoji DNA *via* formation of **T**:B-S^c^ triads and either **T**:P-Z, **C**:P-Z, or **K**^**n**^:P-Z triads, respectively.

EMSA analysis at pH 7.0 confirmed that all TFO constructs successfully assembled into discrete, slower-migrating species in a concentration-dependent manner, despite the significant chemical deviation from natural DNA (Supplementary Fig. 8). The most stable complexes—those containing **T**:A-T and **Z**:G-C (T1), their 8-amino (T2 and T3) or 2-amino derivatives (T7), as well as **K**^**n**^:8oxG-C triads (T4)—remained intact in tris–acetate running buffer lacking magnesium, indicating particularly robust Hoogsteen complementarity. Successful binding was also observed for sequences harboring **S**^**c**^:8oxA-T (T5) triads or the combination of **T**:ΨU-A and **C**:ΨC-G triads (T6), but required 10 mM magnesium in the running buffer to maintain structural integrity during electrophoresis. Similarly, triplex formation was also evident for all sequences containing Hachimoji base pairs, combining **T**:B-S^c^ with either **T**:P-Z, **C**:P-Z, or **K**^**n**^:P-Z triads. Circular dichroism spectroscopy confirmed that the shifted species corresponded to triplex structures, with all complexes displaying the characteristic enhancement of the negative band near 210 nm, consistent with the adoption of an A-like triplex conformation upon TFO binding (Supplementary Fig. 9)^44^.

UV melting analysis was performed to assess the thermal stability of the resultant complexes (Supplementary Fig. 10, Table 1). Six of the ten triplexes exhibited cooperative melting transitions that shifted to higher temperatures relative to the corresponding duplex-only controls. Notably, the *T*_m_ values for the triplexes incorporating **T**:8amA-T, **Z**:8amG-C, **K**^**n**^:8oxG-C, and **T**:2amA-T triads were almost indistinguishable, clustering at 67 °C. In contrast, the **S**^**c**^:8oxA-T triad was less stable, displaying a substantially reduced *T*_m_ of 47 °C. The remaining four triplexes exhibited a single melting transition that overlapped with the underlying duplex. This was most prominent for Hachimoji constructs containing multiple B-S^c^ and P-Z base pairs, reflecting the high intrinsic stability of the underlying duplex. Importantly, no additional low-temperature transitions were observed for any of these constructs, indicating the dissociation of a single, well-defined complex.

**Table 1:** Melting temperatures and thermodynamic parameters for triplexes containing multiple non-standard base pair combinations at pH 7.0

Seq	Triad(s)	*T*_m_ (°C)	*K*_*D*_ (nM)	Sites (N)	ΔG (kcal/mol)	ΔH (kcal/mol)	-TΔS (kcal/mol)
T0	**T**:A-T / **C**^**+**^:G-C	n.d.	3180 ± 1900	0.86 ± 0.09	−7.50 ± 0.36	−64.5 ± 24.0	57.0 ± 21.4
T1	**T**:A-T / **Z**:G-C	66.4 ± 0.7 (49.2 ± 0.7)	11 ± 1.8	1.06 ± 0.04	−10.8 ± 0.1	−57.7 ± 0.6	46.9 ± 0.6
T2	**T**:8amA-T	67.1 ± 1.0 (43.6 ± 0.5)	26 ± 5.1	0.45 ± 0.01	−10.3 ± 0.1	−64.8 ± 1.1	54.4 ± 1.1
T3	**Z**:8amG-C	67.2 ± 0.9 (38.5 ± 0.4)	17 ± 3.7	0.58 ± 0.01	−10.6 ± 0.1	−66.4 ± 1.0	55.8 ± 1.1
T4	**S**^**c**^:8oxA-T	47.3 ± 0.1 (42.2 ± 0.1)	30 ± 13	0.84 ± 0.02	−10.3 ± 0.3	−27.0 ± 1.1	16.8 ± 0.8
T5	**K**^**n**^:8oxG-C	66.6 ± 1.4 (50.2 ± 2.2)	14 ± 3.1	0.90 ± 0.01	−10.7 ± 0.1	−39.1 ± 0.6	28.4 ± 0.5
T6	**T**:ΨU-A / **C**:ΨC-G	57.6 ± 2.0 (54.1 ± 0.4)	24 ± 5.4	1.09 ± 0.01	−10.4 ± 0.1	−36.2 ± 0.7	25.8 ± 0.6
T7	**T**:2amA-T (1^st^)	66.4 ± 1.1 (53.4 ± 0.1)	2.9 ± 0.3	1.01 ± 0.01	−11.7 ± 0.1	−58.8 ± 0.9	47.1 ± 0.7
T7	**T**:2amA-T (2^nd^)		734 ± 28.0	0.84 ± 0.01	−8.37 ± 0.02	−33.0 ± 1.4	24.6 ± 1.1
T8	**T**:B-S^c^ / **T**:P-Z	67.8 ± 0.6 (66.5 ± 0.2)	12 ± 2.4	0.92 ± 0.05	−10.8 ± 0.1	−49.4 ± 0.6	38.6 ± 0.6
T9	**T**:B-S^c^ / **C**:P-Z	66.5 ± 0.9 (66.5 ± 0.2)	15 ± 2.1	0.73 ± 0.04	−10.7 ± 0.1	−43.5 ± 0.4	32.8 ± 0.4
T10	**T**:B-S^c^ / **K**^**n**^:P-Z	69.0 ± 1.5 (66.5 ± 0.2)	23 ± 3.8	0.81 ± 0.05	−10.4 ± 0.1	−37.8 ± 0.5	27.4 ± 0.4

Melting temperatures in parentheses in column three were obtained from the duplex-only samples.

The thermodynamic parameters for triplex formation were then quantified using isothermal titration calorimetry (ITC) (Fig. 4, Table 1). As a reference, the unmodified triplex sequence containing **T**:A-T and **C⁺**:G-C triads (T0) exhibited weak affinity (*K*_D_ = 3180 ± 1900 nM) and poorly defined binding, as expected at neutral pH (ΔG = −7.50 ± 0.36 kcal mol^−1^) (Supplementary Fig. 11a). Although a large apparent enthalpy was observed, this was almost completely offset by a substantial entropic penalty, consistent with the non-productive or transient association of the oligonucleotide. In stark contrast, replacing the four **C⁺**:G-C triads in this sequence with **Z**:G-C (T1) led to roughly a 300-fold enhancement in TFO affinity (*K*_D_ = 11 ± 1.8 nM) at neutral pH, with a larger ΔG (−10.8 ± 0.1 kcal mol^−1^) driven by a favorable enthalpic contribution (ΔH = −57.7 ± 0.6 kcal mol^−1^).

Across the remainder of the panel, triplex formation was uniformly exergonic, with ΔG values spanning −10.3 to −11.7 kcal mol^−1^ and *K*_D_ values in the 3-30 nanomolar range (Fig. 4). Triads incorporating 8-amino purines (T2 and T3) displayed the most favorable enthalpic profiles (ΔH ≈ −65 ± 1.0 kcal mol^−1^), consistent with additional or strengthened hydrogen bonding interactions in the major groove. However, they exhibited reduced apparent stoichiometries, suggesting partial duplex destabilization arising from the presence of uncompensated amino substituents, consistent with the reduced duplex stability observed by UV melting. Triads incorporating oxidized purines retained productive binding but with attenuated enthalpic driving forces: **S**^**c**^:8oxA-T (T4) exhibited the weakest driving force (ΔH = −27.0 ± 1.1 kcal mol^−1^), while **K**^**n**^:8oxG-C (T5) showed intermediate strength (ΔH = −39.1 ± 0.6 kcal mol^−1^). The triplex containing **T**:ΨU-A and **C**:ΨC-G triads (T6) also displayed comparable thermodynamic stability to these complexes (ΔG = −10.4 ± 0.1 kcal mol^−1^; ΔH = −36.2 ± 0.7 kcal mol^−1^) despite the presence of ~33% pyrimidines in the sequence.

Interestingly, the heat profile obtained for the **T**:2amA-T containing triplex (T7) revealed biphasic binding, indicative of two thermodynamically distinct association events. Increasing the number of injections during the experiment allowed us to resolve these contributions (Supplementary Fig. 11b). The dominant event was the strongest interaction measured in this study (*K*_D_ = 2.9 ± 0.3 nM; ΔG = −11.7 ± 0.1 kcal mol^−1^) and driven by a large favorable enthalpy (ΔH = −58.8 ± 0.9 kcal mol^−1^), consistent with efficient Hoogsteen recognition of duplexes preorganized by 2-amino adenine. The weaker event (*K*_D_ = 734 ± 28.0 nM; ΔG = −8.37 ± 0.02 kcal mol^−1^) likely reflects duplex heterogeneity of the sample, where the presence of the 2-amino on adenine promotes alternative duplex conformations that support a less efficient triplex association.

Finally, targeting Hachimoji DNA through the formation of **T**:B-S^c^ triads and either **T**:P-Z, **C**:P-Z, or **K**^**n**^:P-Z generated stable complexes with *K*_D_ values of 12–23 nM and ΔG values clustered between −10.4 and −10.8 kcal mol^−1^. These interactions were consistently enthalpy-driven, although with smaller ΔH values than those observed for the standard or amino purines, suggesting that the steric and/or electronic features of the triads were less optimized.

Collectively, these data establish that modular combinations of modified triads enable stable, well-defined triplex formation across chemically diverse duplex targets. Triplex stability is primarily governed by favorable enthalpic contributions arising from optimized Hoogsteen interactions. These thermodynamic trends correlate closely with the relative stabilities inferred from melting analysis, providing a mechanistic framework for future base triad designs.

### Recognition of 8oxG in enzymatically assembled constructs

8oxG is one of the most prevalent DNA lesions in genomic DNA and is highly mutagenic, commonly pairing with adenine during replication to generate GC → TA transversions^45^. The ability to distinguish 8oxG-C from the mispair 8oxG-A or any other W-C base pair, within long DNA constructs, is of strong desire. We therefore investigated the interactions of **K**^**n**^ with base pair mismatches that might be introduced through replication errors by DNA polymerase, as well as with 8oxG lesions introduced into plasmids or PCR products. Experiments were undertaken at pH 7.4 to better reflect physiological conditions.

**Fig. 5:**
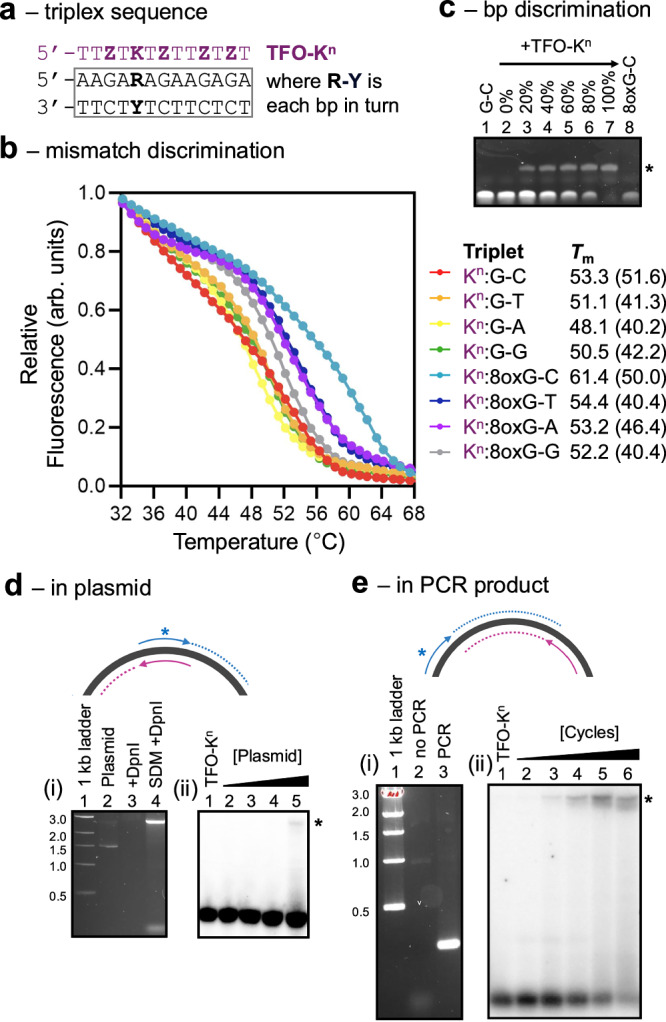
Targeting 8oxG-C in synthetic and enzymatically assembled constructs. **a** Triplex motif is used to assess the interaction of **K**^**n**^ with mismatched G-Y and 8oxG-Y base pairs, where Y is each base in turn. Oligonucleotides were annealed in a pH 7.4 sodium cacodylate buffer with 10 mM magnesium at a final duplex and TFO concentration of 1 μM and 2 μM, respectively. **b** Fluorescence melting profiles. Samples were melted at 0.2 °C min⁻¹ in the presence of SYBR Green I (Ex 488 nm/Em 522 nm. Melting temperatures (*T*_m_) were determined, with **K**^**n**^:8oxG–C triad exhibiting the highest thermal stability. *T*_m_ values for duplex-only samples are shown in parenthesis. **c** Representative electrophoretic mobility shift assay for TFO-**K**^**n**^ with samples containing increasing proportions of 8oxG-C base pairs (*n* = 2). Complexes were separated on a 20% non-denaturing polyacrylamide gel in standard tris-acetate running buffer and subjected to post-staining with GelRed. Triplex formation was evidenced by reduced mobility of a sample relative to the duplex only control (black asterisk) and increased as the percentage of 8oxG increases. **d** 8oxG–C was introduced into pUC18 by site-directed mutagenesis, the parental DNA digested by DpnI (i), and probed with 5′-³²P-labeled TFO-**K**^**n**^ (ii) at reducing plasmid concentrations (*n* = 2). Complexes were resolved on a 12% native polyacrylamide gel in tris–acetate buffer, and the TFO-bound sample was visualized by phosphorimaging (black asterisk). **e** An 8oxG–C lesion was introduced into a 251-bp fragment by PCR and targeted with 5′-³²P-labeled TFO-**K**^**n**^ after successive PCR cycles (*n* = 2). Complexes were resolved on an 8% native polyacrylamide gel in tris–acetate buffer, and the TFO-bound sample visualized by phosphorimaging (black asterisk). Source data are provided as a Source Data file.

The selectivity of **K**^**n**^ across relevant base-pairing contexts was investigated using fluorescence melting analysis. Triplexes were assembled with a single **K**^**n**^:G-Y or **K**^**n**^:8oxG-Y triad with a systematic variation of the base at position Y (Fig. 5a). Melting profiles for the matched and mismatched base pairs in the absence of TFO showed that, as expected, G-C and 8oxG-C duplexes were the most thermally stable, followed by 8oxG-A, with the remaining mismatches displaying significantly reduced stabilities (Supplementary Fig. 12b). Analysis of the corresponding triplex melting profiles revealed that the TFO bound all duplex variants; however, the extent of binding and stabilization was highly triad dependent. Notably, the **K**^**n**^:8oxG-C triad was approximately 8 °C more stable than the next most stable triad, **K**^**n**^:G-C or **K**^**n**^:8oxG-T (Fig. 5b). We also assessed the ability of **K**^**n**^ to detect the extent of oxidation in duplex constructs by assaying its interaction with samples containing different ratios of 8oxG-C to G-C base pairs by EMSA analysis (Fig. 5c). As expected, as the percentage of 8oxG-C increases, the extent of triplex formation increases.

We next assessed whether TFO-**K**^**n**^ could target a triplex-forming sequence containing a single 8-oxoG lesion embedded within longer DNA fragments assembled by primer extension. These experiments used a modified pUC18 plasmid containing the same triplex target sequence used above (T1)^19^. A 31-mer mutagenic primer was designed to substitute the central A-T base pair with an 8oxG-C pair upon extension by Taq polymerase, thereby recreating the same local sequence context as the synthetic 8oxG-C duplex also used above (Supplementary Table 5). Following thermal cycling, the parental plasmid was digested with DpnI, and the resulting linear product (Fig. 5di) was probed with 5′-radiolabelled TFO-**K**^**n**^. Separation by PAGE revealed concentration-dependent interactions; however, binding was only detectable at the highest plasmid concentration tested, owing to the low yield of plasmid generated under these non-PCR conditions (Fig. 5dii). Equivalent experiments were undertaken with a non-mutagenic primer and probing with TFO-**K**^**n**^ revealed, as expected, no interaction of the oligonucleotide (Supplementary Fig. 13b).

To improve the sensitivity of detection, a PCR-based amplification strategy was then implemented using the same mutagenic primer in combination with a second primer located approximately 250 bp upstream in the plasmid. The resulting product was assayed as a function of PCR cycle by probing with 5′-radiolabelled TFO-**K**^**n**^ (Fig. 5d). Under these exponential amplification conditions, binding was clearly observable after only four PCR cycles and increased progressively with further amplification of the target construct. Equivalent experiments were undertaken with a non-mutagenic primer and probing with TFO-**K**^**n**^ revealed only minimal interaction of the oligonucleotide after 32 cycles (Supplementary Fig. 13c).

Taken together, the data demonstrate that TFOs containing the nucleobase **K**^**n**^ can selectively assay the presence of 8oxoG-C base pairs in both short and long DNA fragments assembled enzymatically, establishing a practical route for the detection of this mutagenic lesion in biologically relevant constructs.

### Recognition of AEGIS base pairs in enzymatically assembled constructs

**Fig. 6:**
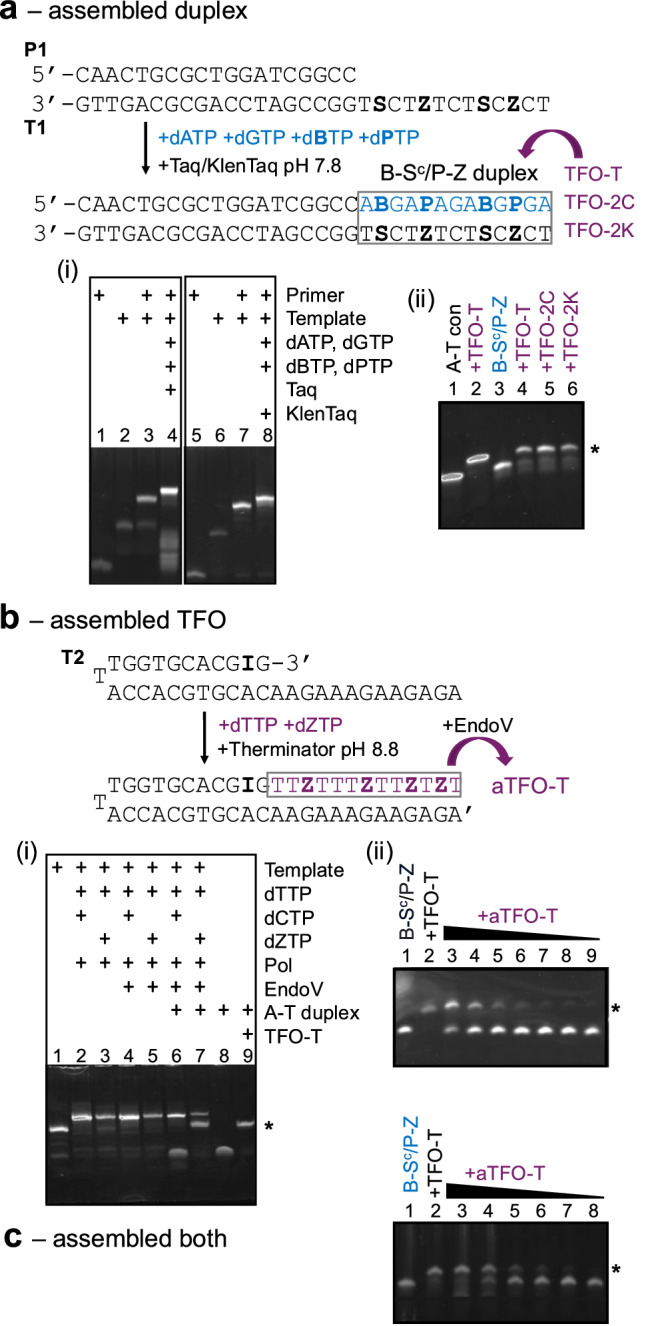
Targeting AEGIS in synthetic and enzymatically assembled constructs. **a** Strategy for assembly of a duplex containing B-S^c^ and P-Z base pairs. Extension reactions were performed for 2 h at 72 °C with 2 units of either Taq or KlenTaq and a final strand and dNTP concentration of 1 μM and 500 μM (dATP, dGTP, and dBTP) or 2500 μM (dPTP), respectively. Representative electrophoretic mobility shift assay (*n* = 2) of (i) the products of extension reactions and (ii) interactions of synthetic TFO-**T**, TFO-**2C**, and TFO-**2K** with the construct assembled using KlenTaq. Complexes were separated on a 20% non-denaturing polyacrylamide gel and subjected to post-staining with GelRed. **b** Strategy for the assembly of a modified TFO capable of binding to B-S^c^ and P-Z. TFO-**T** was assembled and isolated using a modified version of our previous assay^19^. Extension reactions were performed for 2 hrs at 72 °C with 2 units of Therminator DNA polymerase at pH 8.8 and a final strand and dNTP concentration of 1 μM and 500 μM, respectively. Representative electrophoretic mobility shift assay (*n* = 2) of (i) the products of the extension reactions and (ii) interaction of the assembled TFO at a concentration of approximately 250, 100, 25, 10, 25, 1 nM (lanes 3–8, respectively) with the synthetic duplex sequence containing B-S^c^ and P-Z at 1 μM. **c** Combining the products of each reaction from (**a**, **b**) allowed recognition of an enzymatically assembled AEGIS duplex by an enzymatically generated TFO containing AEGIS nucleobases. The assembled TFO was at a concentration of approximately 250, 100, 25, 10, 25, 1 nM (lanes 3-8, respectively) and the assembled duplex containing B-S^c^ and P-Z at approximately 0.25 μM. Source data are provided as a Source Data file.

AEGIS base pairs B-S^c^ and P-Z form part of an eight-letter (“Hachimoji”) genetic alphabet, which is both replicable and transcribable^31^. The ability to recognize assembled duplex sequences containing these base pairs would enable the direct detection and/or functionalization of this synthetic genetic information. Earlier in this study, we established that **T** allows stable recognition of B-S^c^, while **T,** **C**, or **K**^**n**^ can each be used to recognize P-Z through stable Hoogsteen interactions. We therefore sought to determine if these modified TFOs could target a Hachimoji DNA sequence assembled enzymatically (Fig. 6).

To assemble the modified construct, we utilized a simple primer extension assay to generate the same Hachimoji duplex containing B-S^c^ and P-Z base pairs used above (T8-T10) (Fig. 6a). The template was designed to encode the modified pyrimidine strand, with its purine complement assembled from a mixture of standard and AEGIS nucleotide triphosphates (Supplementary Table 6). Primer and template strands were annealed and subjected to polymerase extension at 72 °C in the presence of the four natural dNTPs together with dBTP and dPTP. Because incorporation of dPTP opposite **Z** is known to be influenced by pH^46,47^, these reactions were performed at pH 7.8 and with an excess of dPTP to promote efficient incorporation into the extended duplex.

Extension reactions were carried out using either Taq polymerase, which exhibits limited tolerance for unnatural nucleotides, or KlenTaq polymerase, which lacks 3′–5′ exonuclease proofreading activity and has previously been shown to be more permissive towards modified substrates. Reaction products were analyzed by EMSA (Fig. 6ai). In both cases, polymerase extension was evident, yielding fragments longer than the primer-template duplex with reduced electrophoretic mobility, consistent with successful incorporation of AEGIS nucleotides. However, reactions performed with Taq polymerase also produced a population of shorter, aborted extension products, indicative of reduced processivity on the modified template. Subsequent experiments were therefore conducted exclusively using KlenTaq to maximize yield and homogeneity of the AEGIS-containing duplex substrates used for triplex targeting studies.

Having demonstrated successful assembly of the Hachimoji duplex, we then investigated whether it could be targeted by the synthetic TFOs that position **T** opposite B-S^c^ and either **T,** **C**, or **K** opposite P-Z (Fig. 6aii). In all cases, triplex formation was observed, as evidenced by the formation of slower migrating species that ran at an identical position on the gel as an equivalent synthetic A-T control duplex targeted by TFO-**T**.

We next asked whether an enzymatically assembled TFO could target the synthetic AEGIS-containing duplex substrate studied above (T8–T10; Fig. 6b). To this end, we assembled the TFO-**T** oligonucleotide designed to generate **T**:B-S^c^ and **T**:P-Z triads, using a modified primer-extension protocol previously developed for the synthesis of **ZT**-containing TFOs by exploiting Z-G pairing under high pH conditions^19^. The protocol was adapted to enable release of the completed TFO after synthesis through incorporation of an inosine-containing cleavage sequence compatible with EndoV digestion^48^. Successful assembly and release of the oligonucleotide was confirmed by first demonstrating triplex formation with a standard A-T duplex target, yielding the expected shifted species by PAGE (Fig. 6bi, lane 7). Using this validated approach, the enzymatically assembled TFO was then incubated with the synthetic Hachimoji duplex and analyzed by EMSA, revealing clear, concentration-dependent binding to the AEGIS-containing target (Fig. 6bii).

Finally, we examined whether an entirely enzymatic workflow could be implemented by combining the enzymatically assembled TFO with the enzymatically assembled AEGIS duplex construct. Both components were prepared independently using the corresponding polymerase-driven protocols and subsequently incubated together. Once again, a distinct concentration-dependent triplex species was observed by PAGE analysis, demonstrating successful triplex formation (Fig. 6). Collectively, these experiments establish that both the recognition element and the target duplex can be generated enzymatically while retaining full triplex-binding functionality, thereby enabling a fully polymerase-compatible interface for the assembly and recognition of triplexes containing an expanded genetic alphabet.

## Discussion

DNA recognition by parallel triplex formation is governed by specific Hoogsteen hydrogen bonding patterns within the duplex major groove^13,14^. By systematically probing and reprogramming these interactions using nucleobases from an artificially expanded genetic information system, we have established a partially orthogonal recognition code that operates efficiently under physiologically relevant conditions. Rather than a strict one-to-one mapping, this code is defined by preferential, yet not exclusive, interactions, enabling nucleobases to recognize multiple given base pairs with varying affinity and selectivity. The modular nature of this system allows nucleobases to be combined and deployed interchangeably to target duplex sequences containing diverse arrangements of standard, damaged, and synthetic base pairs, while maintaining nanomolar affinity across highly modified targets.

**Fig. 7:**
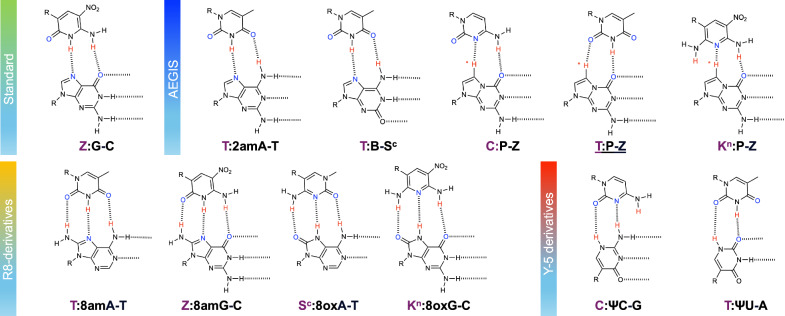
Putative triad structures formed with standard and synthetic base pairs. Chemical structures of possible triad pairings formed through the interaction of each third strand nucleobase (X) with the Hoogsteen face of the recognized duplex base pair (R-Y). All form through established geometries except for the underlined T:P-Z triplet.

Interactions of the nucleobases largely follow predictable geometric and hydrogen bonding rules based on shape complementarity and donor-acceptor positioning (Fig. S2). Recognition of purines typically involves interactions between the 3- and 4-positions of the third strand nucleobase and the 7- and 6-positions of the purine within the base pair^13,14^. Conversely, recognition of pyrimidines generally involves the 2- and 3-positions of the third strand base and the 5- and 4-positions of the pyrimidine^49,50^. Consistent with these principles, of the 22 stable triads examined, 16 appear to conform to these established geometries and included the **Z**:8amG-C**, Z**:G-C, **T**:8amA-T, **T**:2amA-T, **T**:A-T, **T**:B-S^c^**, C**:ΨC-G, **C**^+^:8amG-C, **K**^**n**^:8oxG-C, **C**:P-Z, **K**^**n**^:P-Z, **S**^**c**^:8oxA-T, **Z**:ΨU-A, C:G-C, **T**:ΨU-A, **C**8oxG-C triads (Fig. 7, Supplementary Fig. 14). The use of such triads in combination is likely to minimize backbone distortion of both the third strand and duplex at junctions between adjacent triads.

Notably, deviations from these geometries are observed, reflecting the inherent flexibility of Hoogsteen recognition within the major groove. These likely reflect alternative bonding that involves the 2- and 3-positions of the third strand base and a purine partner, or 3- and 4-positions of the third strand base and a pyrimidine partner, and included **C**:ΨU-A, **Z**:P-Z, **K**^**n**^:B-S, **K**^**n**^:ΨU-A, **T**:P-Z, **V**:G-C triads (Fig. 7, Supplementary Fig. 14). While the formation of these triads at isolated positions within a triplex is noteworthy, their lack of isostructural compatibility with canonical T:A-T, C^+^:G-C and/or Z:G-C triads is likely to limit their use in multiply modified TFO architectures.

Most notably, **V**, which was anticipated to bind A-T, instead preferentially recognized a G-C base pair (Supplementary Fig. 4a). This preference is particularly intriguing and might be explained by a requirement to compensate for the 2-amino group and reduce electrostatic repulsion or unfavorable desolvation penalties^32^. However, **S**^**c**^ and **K**^**n**^ also possess 2-amino groups and did not form equivalent triad geometries with A-T base pairs in this configuration. This hypothesis could be tested using 5-nitro uridine, which more closely mirrors **T** while retaining the nitro group. A second possibility is that the 5-nitro substituent on **V** may perturb the triad’s electronic structure in a way that specifically disfavors A-T pairing. For example, steric interactions with the adjacent 4-carbonyl may force the nitro group out of plane, decreasing the acidity and positioning of the ring N-H group, weakening hydrogen-bond donation. This contrasts with **Z** and **K**^**n**^, which also contain 5-nitro groups but possess adjacent 4-amino substituents capable of intramolecular hydrogen bonding that likely enforce planarity and enhance hydrogen-bonding interactions (e.g., Supplementary Fig. 15).

Across the panel, no simple correlation between hydrogen bond number and triad stability was observed, consistent with a partially orthogonal recognition system in which stability emerges from a combination of geometric complementarity, electronic effects, π-stacking, and steric constraints rather than hydrogen bonding alone. EMSA and melting analysis revealed that the most stable triads generated with purines involved **Z** opposite G-C or 8amG-C, followed closely by **T** opposite A-T, 8amA-T, 2amA-T, or B-S^c^. Intermediate stability was observed for **K**^**n**^ opposite 8oxG-C and **C** and **K**^**n**^ opposite P-Z, with further reductions in stability for **S**^**c**^ opposite 8oxA-T and **T** opposite P-Z. As expected, all but the least stable triad **T**:P-Z form conventional triad geometries that, due to their isostructural nature, will limit distortions to the triplex backbone upon assembly. ITC measurements corroborated this hierarchy and highlighted that the **Z**:8amG-C, **T**:8amA-T, and **T**:2amA-T triads, each capable of additional hydrogen-bonding interactions on either the Hoogsteen or Watson–Crick face, were the most stable of the triads studied. Previous work by Orozco and co-workers demonstrated the stabilizing effects of **C⁺**:8amG-C and **T**:8amA-T triads within pH-dependent triplex motifs^37,51^; here, we extend this strategy to neutral pH through the use of pH-independent **Z**:8amG-C and/or **Z**:G-C triads.

The most stable triad generated with pyrimidines arose from interactions of **C** **K**^**n**^, **Z**, and **T** opposite ΨU-A, and of **C** opposite ΨC-G. Combining **T**:ΨU-A and **C**:ΨC-G enabled triplex recognition of a mixed target sequence containing 33% pyrimidines. Although related strategies have been reported previously^39^, combining these triads with the **Z**:G-C triad markedly enhances triplex stability at neutral pH and, in principle, provides a means to regulate the expression of synthetic genes incorporating these base pairs^8^.

The ability of **K**^**n**^ to detect 8oxG lesions is particularly significant, representing the first demonstration of stable triplex recognition of an oxidative lesion under neutral pH conditions. **K**^**n**^ was prioritized over its non-nitro counterpart to minimize the likelihood of N3 protonation at neutral pH^52^. Consistent with this, experiments performed at pH 5.0 revealed only partial protonation of the nucleobase, as evidenced by the formation of a **K**^**n**^:8amG-C triad. Moreover, melting analysis showed that a single **K**^**n**^:8oxG-C triad is approximately 8 °C more stable than the next most stable triads formed with G-C or the 8oxG-A base pair mismatch. Selectivity of the nucleobase is retained with long, enzymatically assembled DNA fragments, establishing a non-destructive strategy for the direct detection of oxidative damage in biologically relevant constructs.

One of our original motivations for integrating AEGIS nucleobases into triplex DNA—either in the third strand or within the duplex—is their inherent compatibility with polymerase-mediated assembly. As a proof-of-principle, we show that Hachimoji duplex sequences generated from modified nucleotide triphosphates can be targeted by synthetic TFOs as well as by TFOs produced enzymatically. This capability opens several avenues. First, because AEGIS-containing sequences do not cross-pair with natural DNA, they prevent unintended priming in complex genomic samples, enabling fully orthogonal amplification architectures in which TFO-based molecular beacons could act as readers of this amplified information. Second, oligonucleotides containing AEGIS nucleobases have been used extensively to construct aptamers and non-standard DNA architectures^53^, and TFOs provide a means to fold or functionalize such structures^4^. Third, the biological compatibility of AEGIS triplexes creates a feasible path toward coupling TFO assembly with the regulation of natural or artificial genes constructed from an expanded genetic alphabet.

These observations define current design constraints for the expanded triplex recognition code. In particular, two key challenges remain. The stability of triads formed with P-Z is currently limited by suboptimal hydrogen bonding, likely arising from weak C–H…N interactions between the 3-position of the third stand base and 7-position of P^35^. Derivatives of P that address this imbalance are therefore desirable. Targeting mixed pyrimidine/purine sequences also remains difficult because T, S^c^, and Z introduce bulky substituents into the major groove that destabilize triplex formation. Potential solutions include replacing T with pseudouridine, as shown in this study; substituting S^c^ with isocytosine or pseudocytosine analogs^54^, to remove the methyl group from the major groove; and/or developing alternative variants of Z that retain pH-independent G-C recognition while reducing steric effects^47,55^.

The next phase of this work will focus on structural characterization of the modified base triads to define their precise hydrogen-bonding and stacking geometries. These insights will establish a mechanistic framework for the rational design of next-generation triplex-forming nucleotide analogues, enabling the development of programmable, chemically expanded recognition systems for applications in molecular medicine and synthetic biology.

## Methods

### Oligonucleotides

The sequence of each of the unmodified and modified oligonucleotides used in this study is shown in Supplementary Tables 2-6. Unmodified oligonucleotides were purchased from Sigma Aldrich (UK), oligonucleotides containing the synthetic nucleobases 8oxA, 8oxG, 8amA, 8amG, ΨC, ΨU, 5ohC, 5ohU, 2amA, and 5NI were purchased from ATDbio (UK), while oligonucleotides containing the synthetic nucleobases **Z** **S**^**c**^, **K**^**n**^, **V**, B, B^c^, and P were synthesized by Firebird Biomolecular Sciences (US).

### Nucleotide triphosphates

Unmodified deoxyribonucleotide triphosphates were purchased from Promega (UK), whilst modified deoxyribonucleotide triphosphates of **Z**, B, and P were synthesized by Firebird Biomolecular Sciences (US).

### Electrophoretic mobility shift assay (EMSA)

Interactions of the TFOs with duplexes containing standard and non-standard base pairs were determined by native EMSA at different pH. Oligonucleotides were prepared in 10 mM sodium cacodylate containing 10 mM magnesium chloride at either pH 5.0, 7.0, or 9.0 as indicated by the experiment. The final duplex concentration was 1 μM, and the final TFO concentration varied between 30 μM and 0.01 μM in a total volume of 20 µL. Duplex strands were annealed by heating the strands at 95 °C for 5 min and slowly cooling the samples to room temperature over 2 h. The TFOs were then added to the annealed complexes and left to equilibrate for greater than 16 h at 4 °C. Samples were subjected to non-denaturing polyacrylamide gel electrophoresis at 100 V for ~2 ½ hours in 40 mM tris-acetate running buffer (Sigma Aldrich) at either pH 5.0, 8.3, or 9.0 in the presence or absence of 10 mM magnesium acetate as indicated by the experiment. Gels were post-stained with GelRed (Biotium) and visualized using a Gel Doc XR+ Imaging System (BioRad).

### Fluorescence melting

Thermal melting profiles for the duplexes and triplexes were determined using SYBR Green I (Thermofisher) and either a MyGo Pro or Roche LightCycler^41^. Oligonucleotides were prepared in 10 mM sodium cacodylate containing 10 mM magnesium chloride at pH 7.0. The final duplex concentration was 1 μM and the final TFO concentration was 2 μM in a total volume of 20 µL. Duplex strands were annealed before the addition of the TFOs by heating the strands at 95 °C for 5 min and slowly cooling the samples to room temperature over 2 h. Samples were then left to equilibrate for greater than 16 h at 4 °C, before adding SYBR green I dye at a final working concentration. Fluorescence melting profiles were obtained by recording the emission of SYBR Green I at 522 nm, after an excitation at 488 nm, at a slow ramp rate of 0.2 °C/min between 30 °C and 80 °C. [Continuous temperature change of the LightCycler caps at 0.1 °C/s; however, slower melting profiles were obtained by increasing the temperature by 1 °C/step, leaving the samples to equilibrate over a set period of time.] Melting temperatures (*T*_m_) were determined from the first derivatives of the highest temperature transition in each profile using the analysis software provided by the machines and typically varied by less than 1 °C between replicates. Mean values ± SEM were calculated from three independent measurements on distinct samples.

### Ultraviolet (UV) melting

Thermal melting profiles for the duplexes and triplexes were determined using a Shimadzu UV-3600 UV-Vis-NIR spectrophotometer. Oligonucleotides were prepared in 10 mM sodium cacodylate containing 10 mM magnesium chloride at pH 7.0. The final duplex concentration was 5 μM, and the final TFO concentration was 10 μM in a total volume of 100 µL. Duplex strands were annealed before the addition of the TFOs by heating the strands at 95 °C for 5 min and slowly cooling the complexes to room temperature over 2 h. Samples were then left to equilibrate for greater than 16 h at 4 °C. UV melting profiles were recorded at 260 nm, 1 s response time, in a Shimadzu 8 series micro multi-cell with a 10 mm pathlength between 30 °C and 80 °C at a ramp rate of 0.2 °C/min. Melting temperatures (*T*_m_) were determined from the first derivatives of each profile using the analysis software provided by the machine, and typically varied by less than 1 °C between experiments. Mean values ± SEM were calculated from two independent measurements on distinct samples.

### Circular dichroism (CD)

CD spectra for the duplexes and triplexes were determined using either an Applied Photophysics PiStar-180 or a Chirascan VX spectrophotometer. Oligonucleotides were prepared in 10 mM sodium cacodylate containing 10 mM magnesium chloride at pH 7.0. The final duplex concentration was 5 μM, and the final TFO concentration was 10 μM in a total volume of 300 µL. Duplex strands were annealed before the addition of the TFOs by heating the strands at 95 °C for 5 min and slowly cooling the complexes to room temperature over 2 h. Samples were then left to equilibrate for greater than 16 h at 4 °C. Spectra were collected between 320–200 nm, at 100 nm/min, 1 s response time, 1 nm bandwidth in Hellma synthetic quartz cuvettes with a 1 mm pathlength. Each spectrum was accumulated five times and averaged to smooth.

### Isothermal titration calorimetry (ITC)

Thermodynamic parameters for each of the triplexes were determined using a MicroCal PEAQ-ITC Auto (Malvern Panalytical). Oligonucleotides were prepared in 10 mM sodium cacodylate containing 10 mM magnesium chloride at pH 7.0. The calorimeter cell held the duplex target at 5 μM in a total volume of 200 µL, while the syringe contained the TFO binding partner at a concentration of 60 μM. Titration experiments were performed at 25 °C at a reference power of ~9.5 μcal/s. Unless otherwise stated, nineteen aliquots of 4 µL were injected from the syringe at 2.5 min intervals with syringe screw-paddle agitation at 750 rpm. The binding isotherms were processed, and the thermodynamic parameters were obtained using the PEAQ-ITC analysis software v1.52. Residual heat of dilution produced from injecting the TFO was automatically offset by the software and validated by TFO-into-buffer controls.

### Site-directed mutagenesis (SDM)

An 8-oxoG lesion was introduced into a triplex target sequence previously cloned in pUC18 using a modified version of the Agilent® QuickChange site-directed mutagenesis protocol. The 31-mer mutagenic forward primer carried a single 8oxG residue that, upon polymerase extension, replaced a single adenine in the extension product. The overlapping reverse primer contained thymine at the same position to introduce a different substitution on the opposing strand. Reactions contained 20 ng of plasmid DNA, 125 ng of each primer, and 10 mM dNTPs, and were carried out using Taq polymerase (New England Biolabs) in 1X Thermopol® buffer in a final volume of 50 µL. Samples were cycled for 12 rounds in a BioRad® T100 thermal cycler with an initial denaturation at 95 °C for 30 s, followed by annealing at 58 °C and extension at 72 °C. Following assembly, reaction products were digested with 20 units of DpnI for 1 h 30 min at 37 °C to remove parental (methylated) plasmid DNA. Samples were then subjected to a further round of extension to produce the final linearized constructs^56^. A control construct was also prepared using a non-mutagenic forward primer. For triplex binding experiments, the assembled constructs were used directly and diluted 4-, 12-, 40-, 100-, and 400-fold in the assay.

### Polymerase chain reaction (PCR)

A single 8-oxoG lesion was introduced into a triplex target sequence previously cloned in pUC18 using PCR to generate a base-modified 251-bp fragment. The 31-mer mutagenic forward primer carried a single 8oxG residue that, upon polymerase extension, replaced the corresponding adenine in the extension product. A second 31-mer reverse primer was positioned upstream of the sequence to generate the final fragment. The reaction contained 5 ng plasmid, 1 µM forward and reverse primers, 10 mM dNTPs and was performed by Taq polymerase (1.25 units, New England Biolabs) in 1X Thermopol® buffer in a total volume of 50 µL. Samples were cycled for either 2, 4, 8, 16, and 32 rounds in a BioRad® T100 thermal cycler with an initial denaturation at 95 °C for 30 s, followed by annealing at 56 °C and extension at 68 °C. A control construct was also prepared using a non-mutagenic forward primer. For triplex binding experiments, the assembled constructs were used directly.

### Oligonucleotide radiolabelling

To assay TFO binding to enzymatically assembled constructs containing 8oxG, the oligonucleotide carrying a single **K**^**n**^ modification (TFO-**K**^**n**^) was radiolabelled at its 5′-end using T4 polynucleotide kinase (New England Biolabs) and 1 µL [γ-^32^P]ATP (Revvity) in 1X polynucleotide kinase buffer in a total volume of 20 µL for 1 h. The enzyme was then deactivated by heating the sample at 65 °C for 30 min and the oligonucleotide was isolated using a G-25 spin column (Cytiva) to remove excess ATP according to the manufacturer’s instructions. After elution, the oligonucleotide was at a final concentration of *ca*. 0.5 μM in ddH_2_0, yielding approximately 10 c.p.s./µL, as determined by a handheld Geiger counter.

### Duplex assembly by primer extension

The modified duplex sequence containing AEGIS base pairs (B-S^c^/P-Z) was assembled by primer extension of a template containing the appropriate modified nucleobases. Primer and template strands were dissolved in ThermoPol® reaction buffer (New England Biolabs) at pH 7.8 at a final concentration of 1 μM in a total volume of 20 µL. The strands were annealed by heating at 95 °C for 5 min and slowly cooled to room temperature over 2 h. Deoxyribonucleotide triphosphates (*i.e*., dATP/dTTP/dBTP/dPTP) were then added at a final concentration of 500 μM, except for dPTP, which was added at a five-fold excess to promote its incorporation opposite Z^25^. The reaction mixture was pre-incubated at 72 °C for 30 s before the addition of 2 units of either Taq or KlenTaq polymerase (New England Biolabs)^57^. Samples were left >2 h to allow the extension reaction to reach completion. For triplex binding experiments, the assembled duplex was used directly.

### TFO assembly by primer extension

The modified **ZT**-containing TFO (TFO-**T**) was assembled by primer extension on a primer-template containing an inosine residue adjacent to the TFO sequence to enable its release after assembly^19,48^. Primer and template strands were dissolved in ThermoPol® reaction buffer (New England Biolabs) at pH 8.8 at a final concentration of 1 μM in a total volume of 20 µL. Deoxyribonucleotide triphosphates (e.g., dCTP/dTTP or dTTP/dZTP) were then added at a final concentration of 500 μM. The reaction mixture was pre-incubated at 72 °C for 30 s before the addition of 2 units of Therminator® DNA polymerase (New England Biolabs). Samples were left >2 hr to allow the extension reaction to reach completion. EndoV (New England Biolabs) was then used to release the assembled TFO from the extension product by addition of the enzyme (10 units) at 37 °C for 2 h before its inactivation at 80 °C for 10 min. For triplex binding experiments, the assembled TFO was used directly.

### Statistics and reproducibility

No statistical method was used to predetermine sample sizes. These were selected based on standard practice for biochemical and biophysical characterization of synthetic oligonucleotide interactions and are indicated in the relevant figure legends. EMSA experiments used for screening and enzymatically assembled construct analysis were performed at least twice independently, with representative gels shown. Fluorescence melting experiments were performed using three independent measurements on distinct samples, and melting temperatures are reported as mean ± SEM. UV melting experiments were performed using two independent measurements on distinct samples, and melting temperatures are reported as mean ± SEM. CD spectra were accumulated five times for each sample and averaged to improve signal-to-noise. ITC experiments were analyzed using the manufacturer’s software, with residual heats of dilution offset by the software and validated using TFO-into-buffer controls.

No data were excluded from the analyses. The experiments were not randomized. The Investigators were not blinded to allocation during experiments and outcome assessment. Randomization and blinding were not used because the experiments were performed using defined synthetic oligonucleotide sequences and predetermined biochemical conditions rather than biological samples, animals or treatment groups. Reproducibility was assessed by repeating key experiments independently, by analyzing distinct samples where applicable and by comparing orthogonal readouts, including EMSA, fluorescence melting, UV melting, CD spectroscopy and ITC. Source data are provided with the paper, including raw and normalized spectroscopic data and uncropped polyacrylamide gel images.

### Reporting summary

Further information on research design is available in the Nature Portfolio Reporting Summary linked to this article.

## Supplementary information

Supplementary Information

Reporting Summary

Transparent Peer Review file

## Source data

Source Data

## Data Availability

Source data are provided with this paper, including all raw and normalized spectroscopic data and uncropped polyacrylamide gel images. Source data are provided with this paper.
