# Supplementary material for: Recognition of non-standard base pairs by triplex-forming oligonucleotides containing an expanded genetic alphabet
Source: Nat Commun. 2026 Jun 12;17:7477. doi: 10.1038/s41467-026-74375-4 (PMC13407867; doi:10.1038/s41467-026-74375-4)
Supplement: Supplementary file 1 — Supplementary Information [file 41467_2026_74375_MOESM1_ESM.pdf]

Supporting Information

Recognition of Non-standard Base Pairs by Triplex-Forming Oligonucleotides Containing an Expanded Genetic Alphabet

Michael Brazzill,[1] Ruolin Ma,[1] Kieron Munn,[1] Léna Prestifilippo,[1,2] Andrew R. Pickford,[3] Hyo-Joong Kim,[4,5] Cen Chen,[4,5] Shuichi Hoshika,[4,5*] Steven A. Benner,[4,5] and David A. Rusling[1*]

[1]School of Medicine, Pharmacy and Biomedical Sciences, University of Portsmouth, Portsmouth, PO1 2DT, UK

[2]Polytech Angers, 16 Boulevard Daviers, 49100 Angers, France

[3]Centre for Enzyme Innovation, School of the Environment and Life Sciences, University of Portsmouth, Portsmouth, PO1 2DT, UK

[4]Foundation for Applied Molecular Evolution, Alachua, FL 32615, USA

[5]Firebird Biomolecular Sciences LLC, Alachua, FL 32615, USA

*Corresponding authors: david.rusling@port.ac.uk and shoshika@ffames.org

Supplementary Table 1: Nucleobase abbreviations used in this study. Chemical structures of the modified nucleobases are presented in Figure 2 and Supplementary Figure S2.

Abbreviation	Number	Chemical name
**Z**	1	6-amino-5-nitropyridin-2-one
**S^c**	2	1-methylcytosine
**K^n**	3	2,6-diamino-3-nitropyridine
**V**	4	6-amino-3-nitropyridin-2-one
8amA	5	8-aminoadenine
8amG	6	8-aminoguanine
8oxA	7	8-hydroxyadenine
8oxG	8	8-hydroxyguanine
ΨC	9	Pseudoisocytosine
5meC	10	5-methylcytosine
ΨU	11	Pseudouracil
5hoC	12	5-hydroxycytosine
5hoU	13	5-hydroxyuracil
2amA	14	2-aminoadenine
B	15	Isoguanine
P	16	5-aza-7-deazaguanine
B^c	17	7-deazaisoguanine
NI	18	5-nitroindole
I	19	Inosine

Supplementary Table 2: Sequences and naming nomenclature of oligonucleotides used in Figure 3, 5, and Supplementary Figure 6, 7, 11, 12 and 13; numbers in bold indicate nucleobase modifications; underlined bases were those under study.

Name	Oligonucleotide sequences	Modification
Con TFO-**T**	5'-TTCTTTCTTCTCT	
TFO-**T**	5'-TT**1**TTT**1**TT**1**T**1**T	**1** = Z
TFO-**C**	5'-TT**1**TCT**1**TT**1**T**1**T	**1** = Z
TFO-**Z**	5'-TT**1**T**1**T**1**TT**1**T**1**T	**1** = Z
TFO-**S^c**	5'-TT**1**T**2**T**1**TT**1**T**1**T	**1** = Z, **2** = S^c
TFO-**K^n**	5'-TT**1**T**3**T**1**TT**1**T**1**T	**1** = Z, **3** = K^n
TFO-**V**	5'-TT**1**T**4**T**1**TT**1**T**1**T	**1** = Z, **4** = V
Duplex<br>A-T	5'-AAGAAAGAAGAGA<br>3'-TTCTTTCTTCTCT	
Duplex<br>G-C	5'-AAGAGAGAAGAGA<br>3'-TTCTCTCTTCTCT	
Duplex<br>T-A	5'-AAGATAGAAGAGA<br>3'-TTCTATCTTCTCT	
Duplex<br>C-G	5'-AAGACAGAAGAGA<br>3'-TTCTGTCTTCTCT	
Duplex<br>U-A	5'-AAGAUAGAAGAGA<br>3'-TTCTATCTTCTCT	
Duplex<br>8amA-T	5'-AAGA**5**AGAAGAGA<br>3'-TTCTTTCTTCTCT	**5** = 8amA
Duplex<br>8amG-C	5'-AAGA**6**AGAAGAGA<br>3'-TTCTCTCTTCTCT	**6** = 8amG
Duplex<br>8oxA-T	5'-AAGA**7**AGAAGAGA<br>3'-TTCTTTCTTCTCT	**7** = 8oxA
Duplex<br>8oxG-C	5'-AAGA**8**AGAAGAGA<br>3'-TTCTCTCTTCTCT	**8** = 8oxG
Duplex<br>ΨC-G	5'-AAGA**9**AGAAGAGA<br>3'-TTCTGTCTTCTCT	**9** = ΨC
Duplex<br>5meC-G	5'-AAGA**10**AGAAGAGA<br>3'-TTCTGTCTTCTCT	**10** = 5meC
Duplex<br>ΨU-A	5'-AAGA**11**AGAAGAGA<br>3'-TTCTATCTTCTCT	**11** = ΨU
Duplex<br>5ohC-G	5'-AAGA**12**AGAAGAGA<br>3'-TTCTGTCTTCTCT	**12** = 5ohC
Duplex<br>5ohU-A	5'-AAGA**13**AGAAGAGA<br>3'-TTCTATCTTCTCT	**13** = 5ohU
Duplex<br>2amA-T	5'-AAGA**14**AGAAGAGA<br>3'-TTCTTTCTTCTCT	**14** = 2amA
Duplex<br>B-S^c	5'-AAGA**15**AGAAGAGA<br>3'-TTCT**2**TCTTCTCT	**15** = B, **2** = S^c
Duplex<br>P-Z	5'-AAGA**16**AGAAGAGA<br>3'-TTCT**1**TCTTCTCT	**16** = P, **1** = Z
Duplex<br>B*-S^c	5'-AAGA**17**AGAAGAGA<br>3'-TTCT**2**TCTTCTCT	**17** = B^C, **2** = S^c
Duplex<br>Z-P	5'-AAGA**1**AGAAGAGA<br>3'-TTCT**16**TCTTCTCT	**1** = Z, **16** = P
Duplex<br>NI-T	5'-AAGA**18**AGAAGAGA<br>3'-TTCT**T**TCTTCTCT	**18** = NI
Duplex<br>NI-C	5'-AAGA**18**AGAAGAGA<br>3'-TTCT**C**TCTTCTCT	**18** = NI

Supplementary Table 3: Sequences and nomenclature of the oligonucleotides used in Supplementary Figure 4; numbers in bold indicate nucleobase modifications; underlined bases were those under study.

Name	Oligonucleotide sequences	Modifications
TFO-Va	5'-TT**1**TTT**1**TT**1**4**1**T	**1** = Z, **4** = V
TFO-2V	5'-TT**1**TTT**1**T**4**1**4**1T	**1** = Z, **4** = V
TFO-3V	5'-TT**1**TTT**1**4**4**1**4**1T	**1** = Z, **4** = V
Duplex G-C 1	5'-AAGAAAGAAGGGA<br>3'-TTCTTTCTTCCCT	
Duplex G-C 2	5'-AAGAAAGAGGGGA<br>3'-TTCTTTCTCCCCT	
Duplex G-C 3	5'-AAGAAAGGGGGGA<br>3'-TTCTTTCCCCCCT	

Supplementary Table 4: Sequences and nomenclature of the oligonucleotides used in Figure 4, 6, and Supplementary Figure 8, 9, and 10; numbers in bold indicate nucleobase modifications; underlined bases were those under study.

Name	Oligonucleotide sequences	Modifications
Con TFO-T	5'-TTCTTTCTTCTCT	
TFO-T	5'-TT**1**TTT**1**TT**1**T**1**T	**1** = Z
TFO-4S^c	5'-T**21**T**2**T**1**T**2121**T	**1** = Z, **2** = S^c
TFO-4K^n	5'-T**31**T**3**T**1**T**3131**T	**1** = Z, **3** = K^n
TFO-2C	5'-TT**1**TCT**1**TT**1**C**1**T	**1** = Z
TFO-2K	5'-TT**1**T**3**T**1**TT**131**T	**1** = Z, **3** = K^n
Duplex A-T	5'-AAGAAAGAAGAGA<br>3'-TTCTTTCTTCTCT	
Duplex 8amA-T	5'-A**5**GAA**5**GA**5**G**5**GA<br>3'-TTCTTTCTTCTCT	**5** = 8amA
Duplex 8amG-C	5'-AA**6**AAA**6**AA**6**A**6**A<br>3'-TTCTTTCTTCTCT	**6** = 8amG
Duplex 8oxA-T	5'-A**7**GAA**7**GA**7**G**7**GA<br>3'-TTCTTTCTTCTCT	**7** = 8oxA
Duplex 8oxG-C	5'-A**8**GAA**8**GA**8**G**8**GA<br>3'-TCCTTCCTCCCCT	**8** = 8oxG
Duplex ΨU-A / ΨC-G	5'-A**11**GA**9**AGA**11**G**9**GA<br>3'-TACTGTCTACGCT	**9** = ΨC, **11** = ΨU
Duplex 2amA-T	5'-A**14**GAA**14**GA**14**G**14**GA<br>3'-TTCTTTCTTCTCT	**14** = 2amA
Duplex B-S^c / P-Z	5'-A**15**GA**16**AGA**15**G**16**GA<br>3'-T**2**CT**1**TCT**2**C**1**CT	**1** = Z, **16** = P<br>**2** = S^c, **15** = B,

Supplementary Table 5: Sequences and nomenclature of the primers used in Figure 5, and Supplementary Figure 12 and 13; numbers in bold indicate nucleobase modifications; underlined bases were those under study.

Name	Oligonucleotide sequences	Modifications
Mutagenic Forward	5'-GAGCAGCCTAAGA**8**AGAAGAGAGGGACATCA	**8** = 8oxG
Non-mutagenic Forward	5'-GAGCAGCCTAAGAAAGAAGAGAGGGACATCA	
Reverse 1 (SDM)	5'-TGATGTCCCTCTCTTCTTTCTTAGGCTGCTC	
Reverse 2 (PCR)	5'-GCGCGTTGGCCGATTCATT	

Supplementary Table 6: Sequences and nomenclature of the template and primers used in Figure 6; numbers in bold indicate nucleobase modifications; underlined bases were those under study.

Name	Oligonucleotide sequences	Modifications
T1	5'-TC**1**C**2**TCT**1**TC**2**TGGCCGATCCAGCGCAGTTG	**1** = Z, **2** = S^c
P1	5'-CAACTGCGCTGGATCGGCC	
A-T con	5'-CAACTGCGCTGGATCGGCCAAGAAAGAAGAGA<br>3'-GTTGACGCGACCTAGCCGGTTCTTTCTTCTCT	
T2	5'-AGAGAAGAAAGAACACGTGCACCATTGGTGCACG**19**G	**19** = inosine

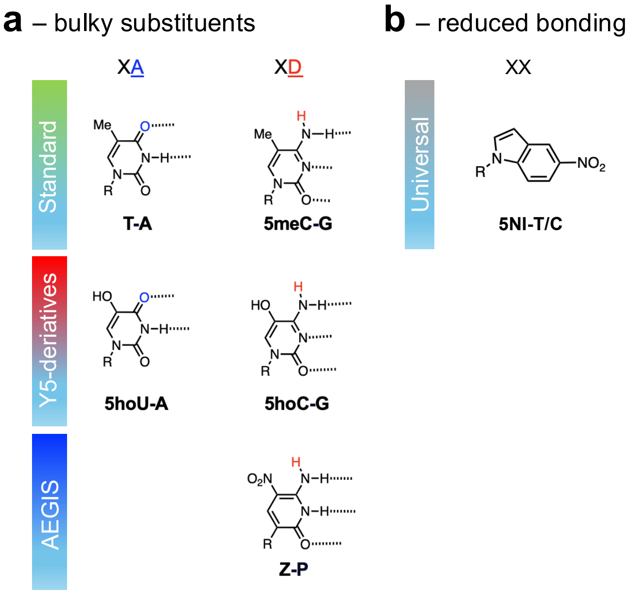

Supplementary Figure 1 – Triplex recognition of natural and non-natural base pairs using an expanded genetic alphabet. (a) Structures of standard and non-standard "pyrimidine" nucleobases (T, 5meC, 5ohU, 5ohC, and Z) presenting a bulky substituent in the major groove. (b) Structure of the universal base analogue 5-nitroindole with reduced Hoogsteen bonding capacity in the major groove. Hydrogen bond donor (D, red) and acceptor (A, blue) arrangements are indicated. Both sets of nucleobases were expected to hinder TFO binding in the major groove and reduce triplex stability.

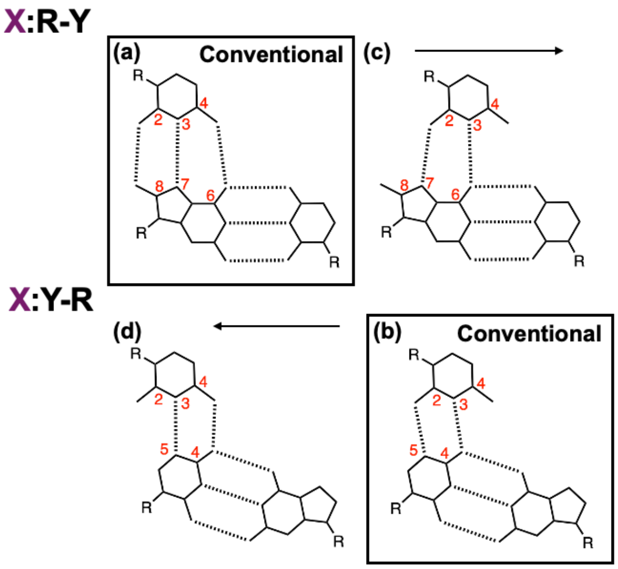

Supplementary Figure 2 – Possible conventional and non-conventional triad interactions for X:R-Y and X:Y-R parallel triads. (a) Recognition of purines typically involves hydrogen bond formation between the 3- and 4-positions of the third strand base and the 7- and 6-positions of the purine within the W-C pair. Recognition of 8-modified purines may allow additional interactions between the 2-position of the third strand base and a group positioned at the 8-position. (b) By contrast, recognition of pyrimidines generally involves bonding between the 2- and 3-positions of the third strand base and the 5- and 4-positions of the pyrimidine. In both cases, unconventional C-H…O/N contacts may contribute additional stability. Other non-conventional pairing geometries are possible that involve a shift in the position of the TFO base further into (c) or out of (d) the major groove. However, the formation of these triads are likely to be destabilising, as they are not isostructural with the **T**:A-T and **Z**:G-C triads formed upon triplex formation.

a – triplex sequence

```
5'-TTCTTTCTTCTCT   where X in TFO-X is T
5'-AAGARAGAAGAGA   where R-Y is each bp in turn
3'-TTCTYTCTTCTCT
```

b – EMSA screening (low pH)

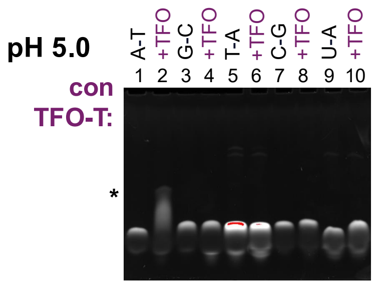

Supplementary Figure 3 – Triplex selectivity of unmodified TFO-T (con TFO-T) at pH 5.0. (a) Triplex motif used to assess nucleobase **(X)** selectivity for each base pair (R-Y) in turn. (b) EMSA analysis of unmodified TFO-**T** at low pH with each duplex. Oligonucleotides were annealed in pH 5.0 sodium cacodylate buffer containing 10 mM magnesium at a final concentration of 1 μM duplex and 2 μM TFO and then separated on a 20% non-denaturing polyacrylamide gel in standard tris-acetate running buffer. As expected, triplex formation was only evident for the triplex generated with a central **T**:A-T triad. Uncropped gel images can be found at the end of the document.

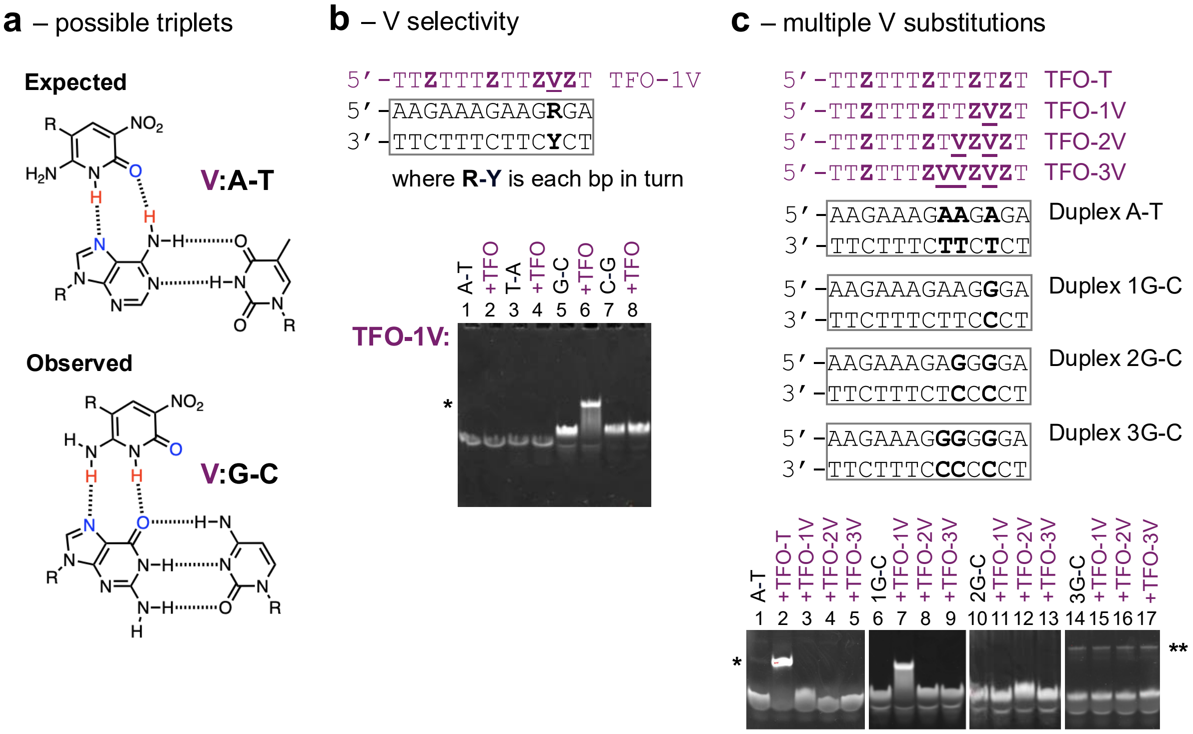

Supplementary Figure 4 – Screening of the V nucleobase in different sequence contexts. (a) Putative structures of the **V**:A-T and **V**:G-C triads under study. (b) Triplex motif used to assess the selectivity of the nucleobase **V** for each base pair (R-Y) in turn within a different sequence context. Oligonucleotides were annealed in pH 7.0 sodium cacodylate buffer containing 10 mM magnesium at a final duplex and TFO concentration of 1 μM and 2 μM, respectively. Samples were then separated on a 20% non-denaturing polyacrylamide gel in standard tris-acetate running buffer. Again, triplex formation is evident for the complex containing an unconventional **V**:G-C triad (black asterisk). (c) Triplex motif used to assess the stability of triplexes containing multiple V:G-C triads by EMSA analysis. Oligonucleotides were annealed in pH 7.0 sodium cacodylate buffer containing 10 mM magnesium at a final duplex and TFO concentration of 1 μM and 2 μM, respectively. Samples were then separated on a 20% non-denaturing polyacrylamide gel in tris-acetate running buffer. As expected, triplex stability decreases as the number of **V**:G-C triads increase, on account of the **V**:G-C triad not being isostructural with **T**:A-T and **Z**:G-C triads. The "*" is used to indicate species that likely correspond to the formation of a triplex, while "**" is used to indicate minor bands present in the duplex-only and triplex samples that likely correspond to unintended interactions between the G-rich or C-Rich duplex strands (Duplex 3G-C). Uncropped gel images can be found at the end of the document.

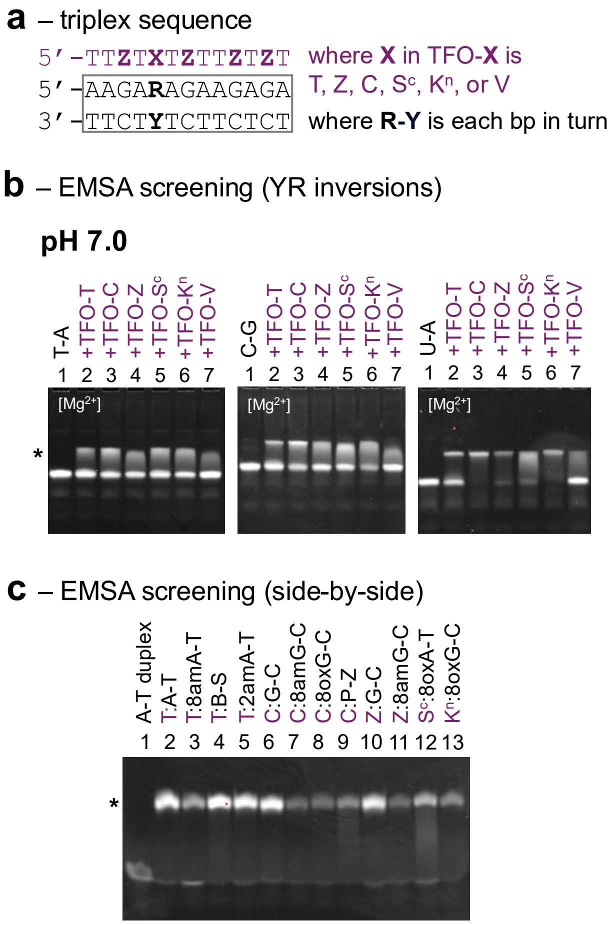

Supplementary Figure 5 – Additional screening of modified TFOs. (a) Triplex motif used to assess nucleobase (**X**) selectivity for each base pair (R-Y) in turn. (b) EMSA analysis of modified TFOs positioning each nucleobase opposite each of the W-C pyrimidine inversions. Oligonucleotides were annealed in pH 7.0 sodium cacodylate buffer containing 10 mM magnesium at a final duplex and TFO concentration of 1 μM and 10 μM, respectively. Samples were then separated on a 20% non-denaturing polyacrylamide gel in standard tris-acetate running buffer containing 10 mM magnesium. Triplex formation with pyrimidine nucleobases is evident under these stabilising conditions, with the most stable triads generated with **C** and **K^n** opposite U-A (black asterisk). (c) EMSA analysis of the major triads formed in Figure 3 but run side-by-side on a single PAGE gel. Oligonucleotides were annealed in pH 7.0 sodium cacodylate buffer containing 10 mM magnesium at a final duplex and TFO concentration of 1 μM and 2 μM, respectively. Samples were then separated on a 20% non-denaturing polyacrylamide gel in standard tris-acetate running buffer containing 10 mM magnesium. Shifted species ran with the same mobility suggesting formation of the same triplex architectures. Uncropped gel images can be found at the end of the document.

a – triplex sequence

5′-TTZTXTZTTZTZT where **X** in TFO-X is C, S^c, K^n

5′-AAGARAGAAGAGA where **R-Y** in duplex is each bp in turn

3′-TTCTYTCTTCTCT

b – EMSA screening at different pH

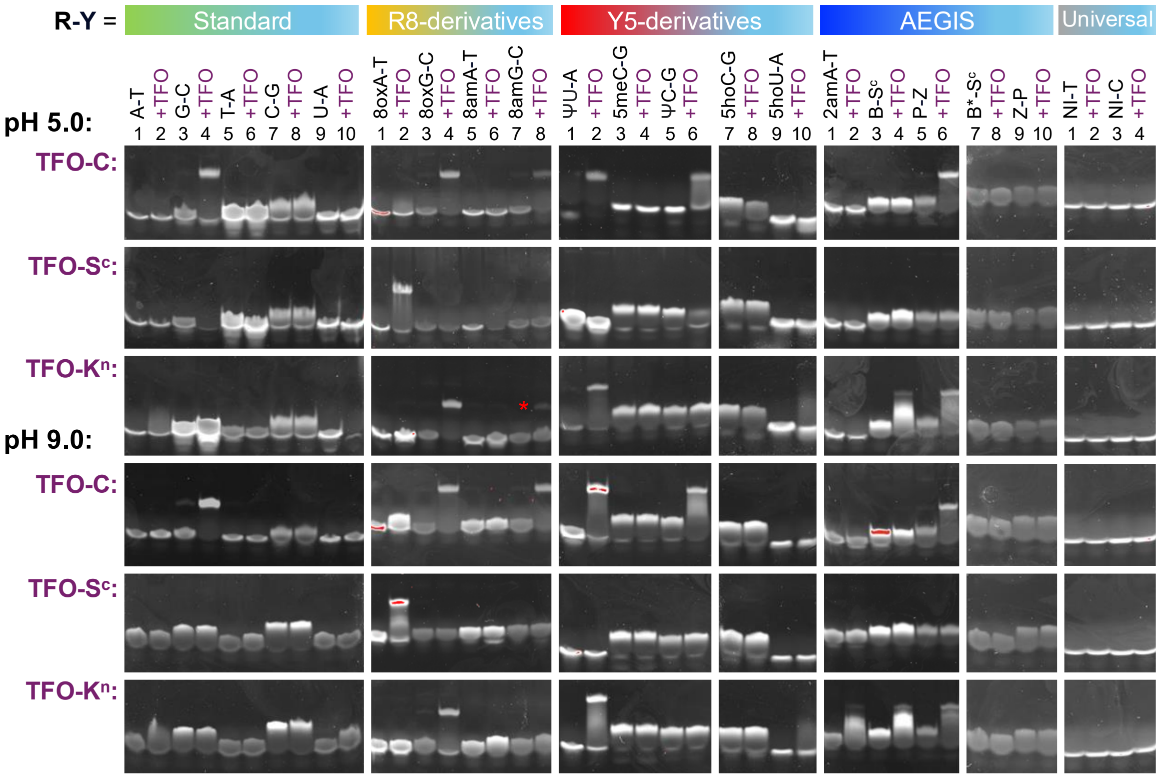

Supplementary Figure 6 – Triplex selectivity of an expanded genetic alphabet (pH analysis). (a) Triplex motif used to assess nucleobase (**X**) selectivity for each base pair (R-Y) in turn at different pH. (b) Electrophoretic mobility shift assay for each sample. Oligonucleotides were annealed in pH 5.0 or pH 9.0 sodium cacodylate buffer containing 10 mM magnesium at a final duplex and TFO concentration of 1 μM and 2 μM, respectively. Complexes were separated on a 20% non-denaturing polyacrylamide gel in tris-acetate running buffer at the equivalent pH and subjected to post-staining with GelRed. Triplex formation is evidenced by reduced mobility of a sample relative to the duplex only control. The selectivity of the three nucleobases remains unaltered at each pH, except for TFO-$\mathbf{K^n}$ which shows a minor interaction with 8amG-C at pH 5.0 indicating minor protonation at N3 (red asterisk). Uncropped gel images can be found at the end of the document.

a – duplex stability (normalisation)

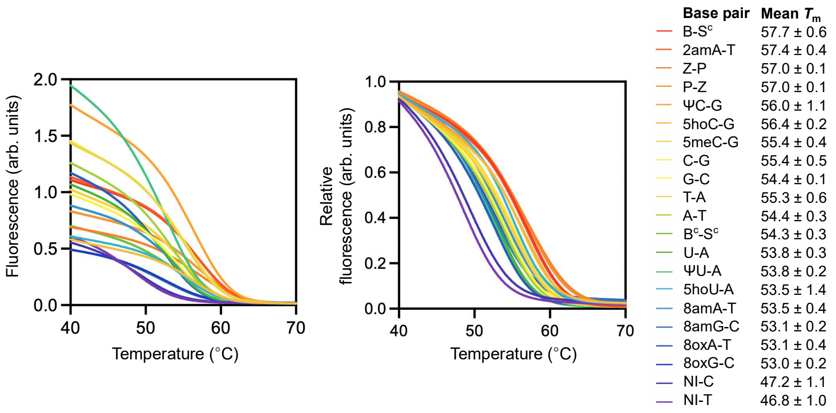

b – triplex stability (normalisation)

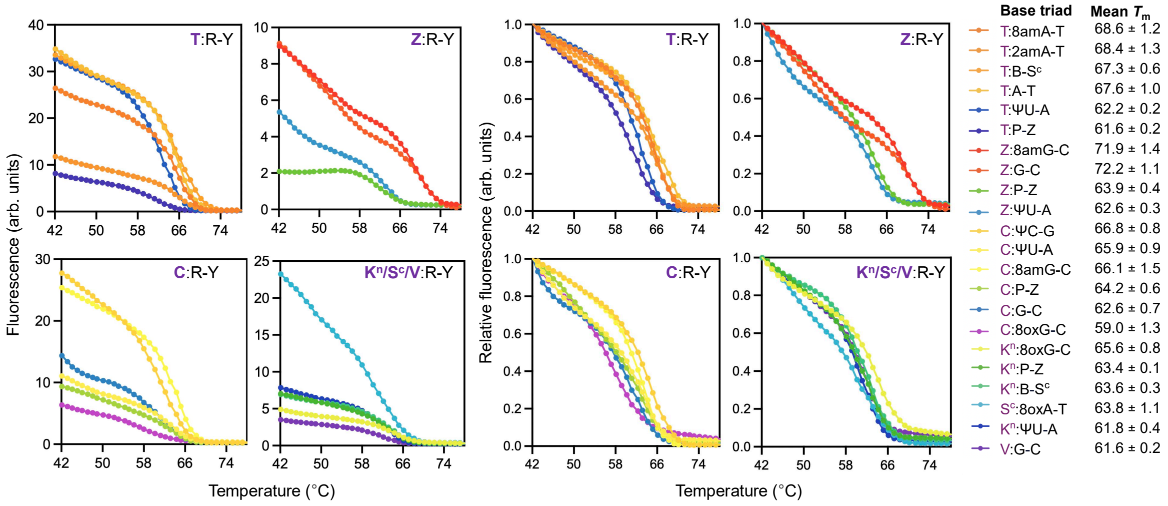

Supplementary Figure 7 – Triplex selectivity of an expanded genetic alphabet (melting analysis). Fluorescence and relative fluorescence melting profiles for the duplex (a) and triplex (b) sequences shown in Figure 3. Oligonucleotides were annealed in pH 7.0 sodium cacodylate buffer containing 10 mM magnesium at a final duplex and TFO concentration of 1 μM and 2 μM, respectively. Samples were then melted at 0.2 °C min^{-1} in the presence of SYBR Green I (Ex 488 nm/Em 522 nm). Duplex and triplex melting temperatures (T_m) were determined from the mid-point of the melting transitions using software provided by the machine and the mean T_m and standard error for each sample determined from three separate replicates. In each case the T_m for each triplex was shifted to higher temperature compared to the equivalent duplex only sample. Thermal stability was dependent on the nature of the triad formed. Source data are provided as a Source Data file.

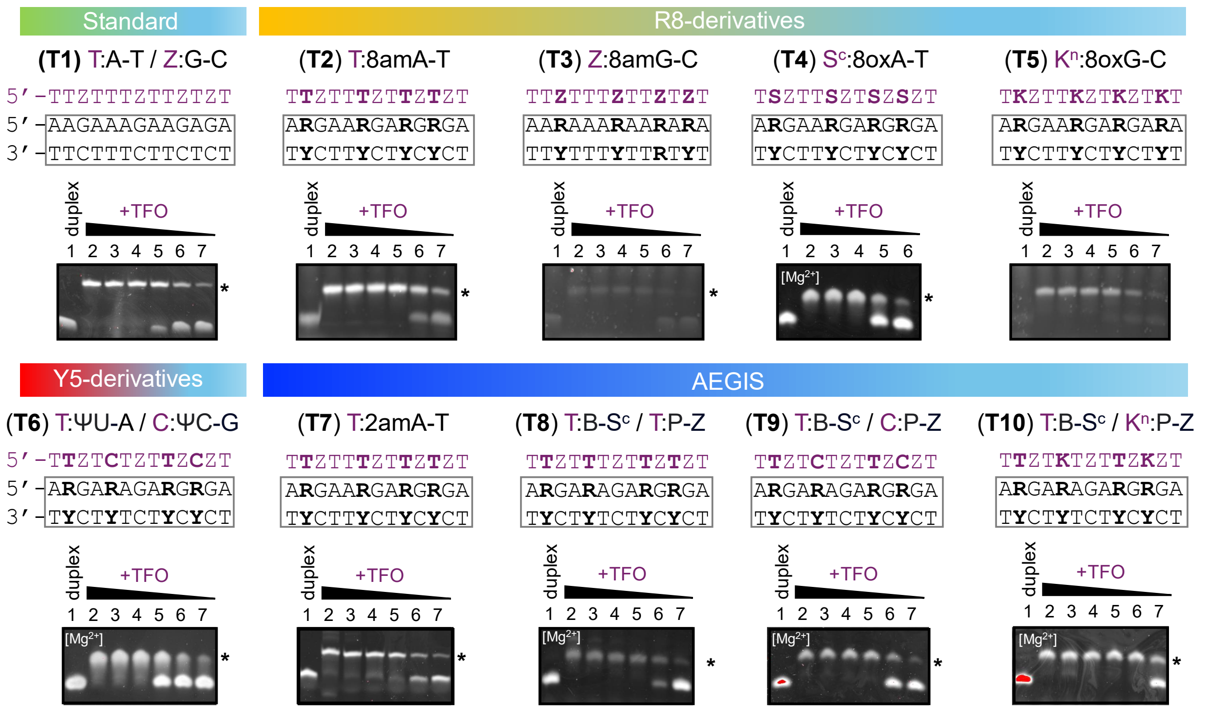

Supplementary Figure 8 – Modular recognition of different base pair combinations (EMSA analysis). Triplex motifs analysed are indicated above each gel and differ in the type and number of modified base triads (T1–T10). Oligonucleotides were annealed in pH 7.0 sodium cacodylate buffer containing 10 mM Mg^{2+}, at a final duplex concentration of 1 μM and TFO concentration of 30, 10, 3, 1, 0.3 and 0.1 μM (lanes 2-7, respectively). Complexes were separated on a 20% non-denaturing polyacrylamide gel in standard tris-acetate running buffer in the presence or absence of 10 mM Mg^{2+} magnesium as highlighted. Gels were then subjected to post-staining with GelRed. Triplex formation was evidenced by reduced mobility of a sample relative to the duplex only control sample and evident for all triplex sequences under study (black asterisk). Sequences T4, T6, T8, T9, T10 required 10 mM magnesium in the running buffer for stability. Uncropped gel images can be found at the end of the document.

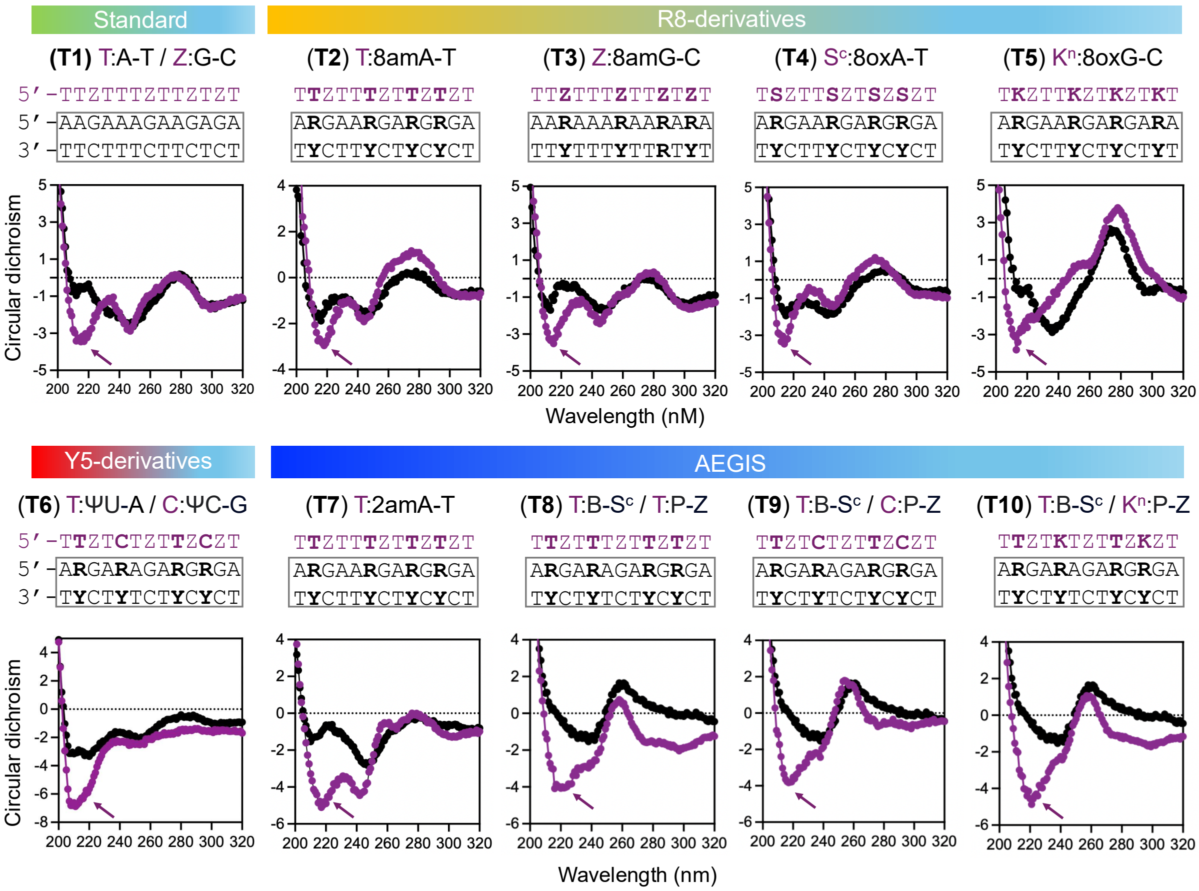

Supplementary Figure 9 – Modular recognition of different base pair combinations (CD spectroscopy). Triplex motifs analysed are indicated above each CD profile and differ in the type and number of modified base triads (T1–T10). Oligonucleotides were annealed in pH 7.0 sodium cacodylate buffer containing 10 mM Mg^{2+}, with final duplex and TFO concentrations of 5 and 10 μM, respectively. Spectra were collected between 320–200 nm, at 100 nm/min, 1 s response time, 1 nm bandwidth in Hellma synthetic quartz cuvettes with a 1 mm pathlength. Each spectrum was accumulated five times and averaged to smooth. Triplex formation (purple data points) was evidenced by an increased negative peak at 210 nm compared to the duplex only sample[1] (black data points) and was observed for all triplex sequences under study (purple arrows). Source data are provided as a Source Data file.

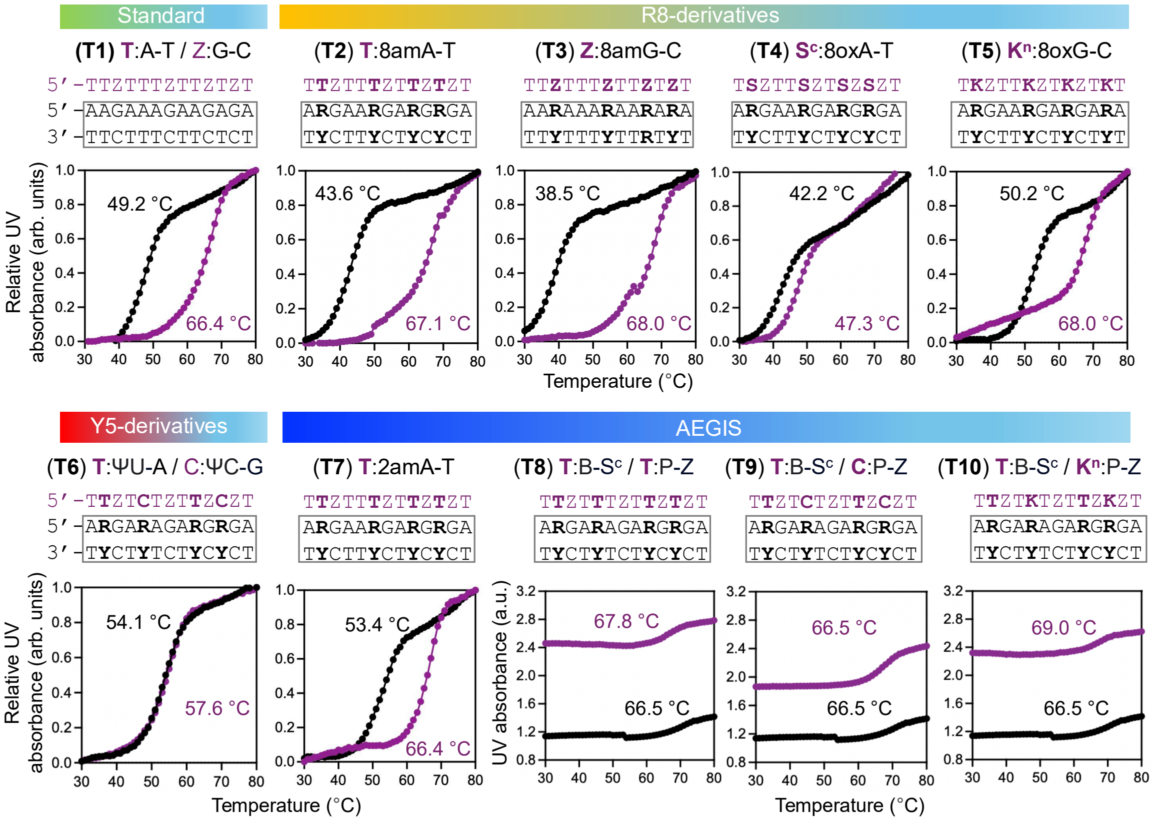

Supplementary Figure 10 – Modular recognition of different base pair combinations (UV melting). Triplex motifs analysed are indicated above each melting profile and differ in the type and number of modified base triads (T1–T10). Oligonucleotides were annealed in pH 7.0 sodium cacodylate buffer containing 10 mM Mg^{2+}, with final duplex and TFO concentrations of 5 and 10 µM, respectively. Samples were heated at 0.2 °C min^{-1} and absorbance monitored using a Shimadzu UV-3600 UV–Vis–NIR spectrophotometer. Melting temperatures (T_m) correspond to the midpoint of the thermal transition. Six triplexes exhibited increased T_m values relative to the corresponding duplex-only controls, indicating enhanced stability. Thermal stability was dependent on the identity of the base triad. Triplexes containing Y5-derivatives and AEGIS base pairs displayed melting profiles comparable to the underlying duplex, consistent with coincident duplex and triplex melting transitions.

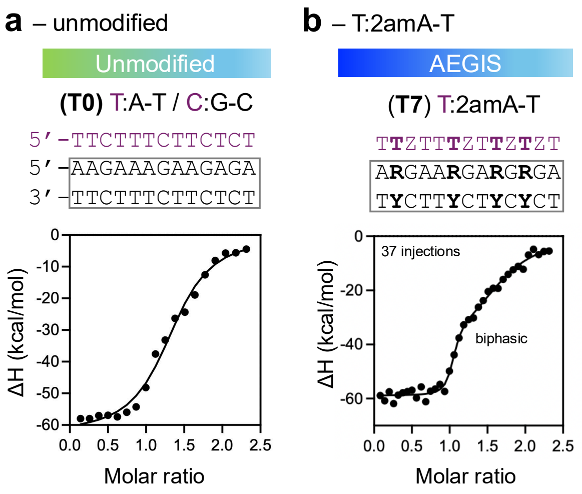

Supplementary Figure 11 – Modular recognition of different base pair combinations (Additional ITC). Oligonucleotides were dissolved in pH 7.0 sodium cacodylate buffer containing 10 mM magnesium at a final duplex and TFO concentration of 5 μM and 60 μM, respectively. (a) The unmodified triplex motif under study is shown (T0). Weak triplex formation is evident with an affinity in the micromolar range (Table 1). (b) The **T**:2amA-T triplex motif under study is shown (T7). Additional injections reveal a biphasic isotherm. Source data are provided as a Source Data file.

a – duplex sequence

5′-AAGA**R**AGAAGAGA
3′-TTCT**Y**TCTTCTCT where **R-Y** is each bp in turn

b – duplex mismatch

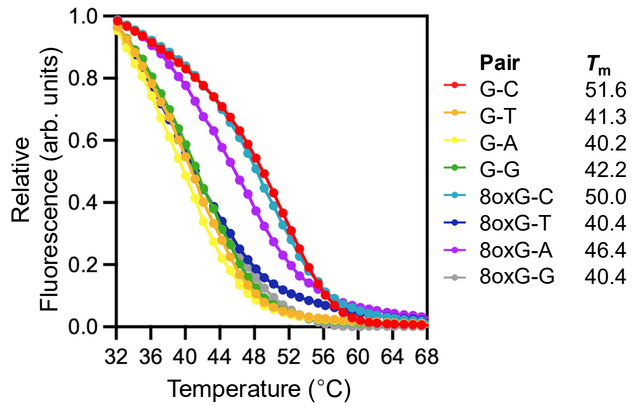

Supplementary Figure 12 – G-Y and 8oxG-Y duplex melting analysis. (a) Duplex motif used to assess the stability of mismatched G-Y and 8oxG-Y base pairs, where Y is each base in turn. (b) Fluorescence melting profiles for the samples. Oligonucleotides were annealed in pH 7.0 sodium cacodylate buffer with 10 mM magnesium at a final concentration of 1 μM and melted at 0.2 °C min^{-1} in the presence of SYBR Green I (Ex 488 nm/Em 522 nm). Melting temperatures (T_m) were determined for each sample, with G-C and 8oxG–C bases exhibiting, as expected, the highest thermal stability. The next most stable was 8oxG-A, then a much lower thermal stability for the remainder of the mismatch combinations. Source data are provided as a Source Data file.

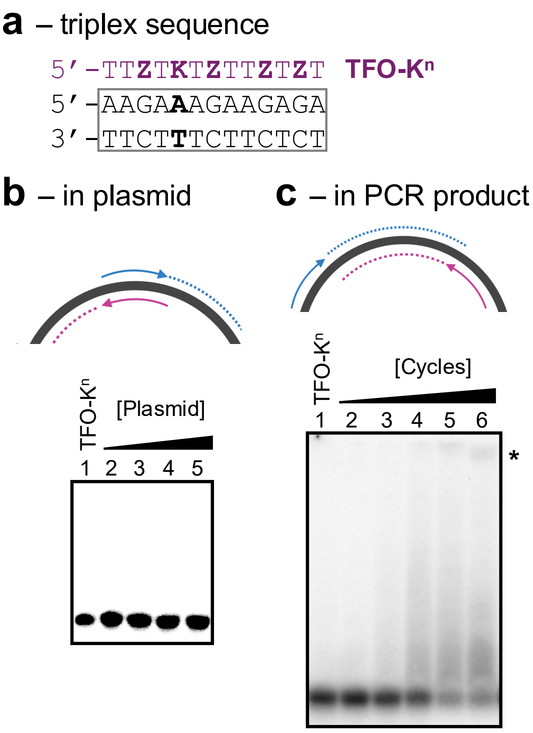

Supplementary Figure 13 – Selectivity of K^n for targeting A-T in enzymatically assembled constructs. (a) Triplex motif used to assess the interaction of **K^n** with an A-T base pair. (b) A-T was introduced into pUC18 by site-directed mutagenesis (SDM, blue asterisk), the parental DNA digested by DpnI, and probed with 5′-^{32}P-labelled TFO-**K^n** at reducing plasmid concentrations. Complexes were resolved on a 12% native polyacrylamide gel in tris–acetate buffer and the TFO-bound sample visualised by phosphorimaging (black asterisk). As observed previously, no binding of **K^n** to A-T was evident under these experimental conditions. (c) A-T was introduced into a 251-bp fragment by PCR and targeted with 5′-^{32}P-labelled TFO-**K^n** after successive PCR cycles. Complexes were resolved on an 8% native polyacrylamide gel in tris–acetate buffer and the TFO-bound sample visualised by phosphorimaging (black asterisk). Only minor binding was observed after 32 PCR cycles (lane 6). Uncropped gel images can be found at the end of the document.

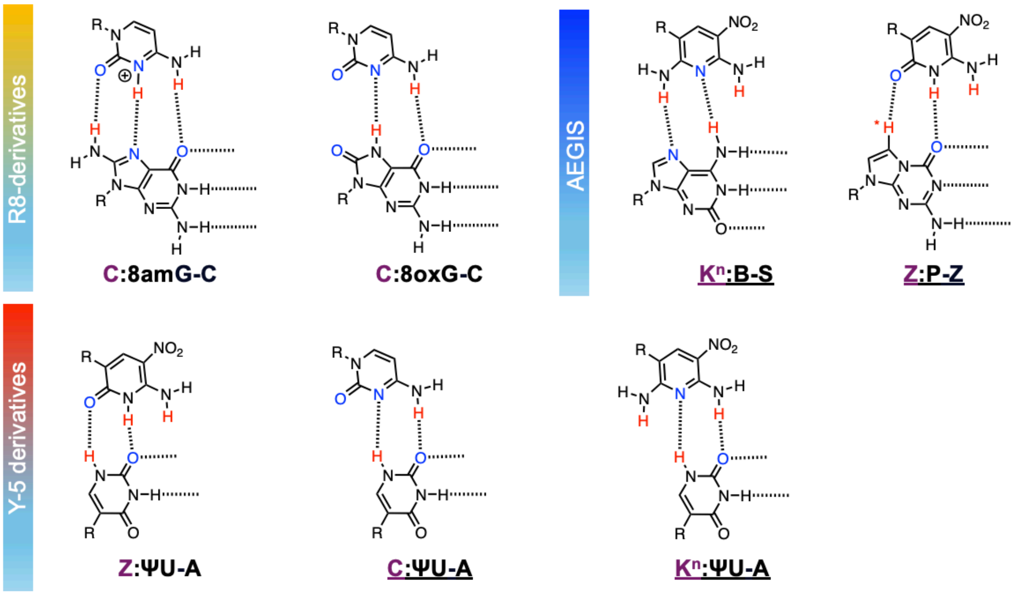

Supplementary Figure 14 – Additional triad structures formed with standard and synthetic base pairs. Chemical structures of possible triad pairings formed through the interaction of each third strand nucleobase (X) with the Hoogsteen face of the recognized duplex base pair (R-Y). Triads underlined are formed through non-conventional triad geometries.

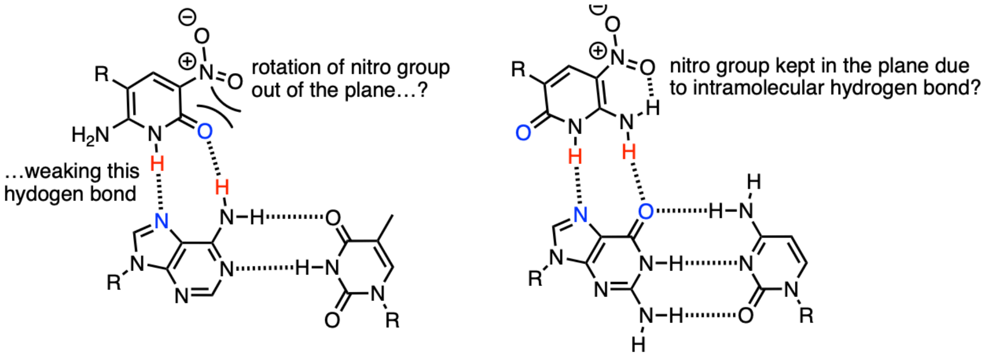

Supplementary Figure 15 – Differential effects of the nitro group in V:A-T (left) and Z:G-C (right) triads. Steric interactions with the adjacent 4-carbonyl may force the nitro group of **V** out of plane, decreasing the acidity and positioning of the ring N-H group, weakening hydrogen-bond donation. By contrast, intramolecular hydrogen bonding between the adjacent 4-amino and nitro group of **Z** likely enforces planarity and enhance hydrogen-bonding interactions.

Uncropped gels in Supplementary Figures 3b, 4b, 4c, 5b, 5c, S8, S13b,

Supplementary Figure 3b

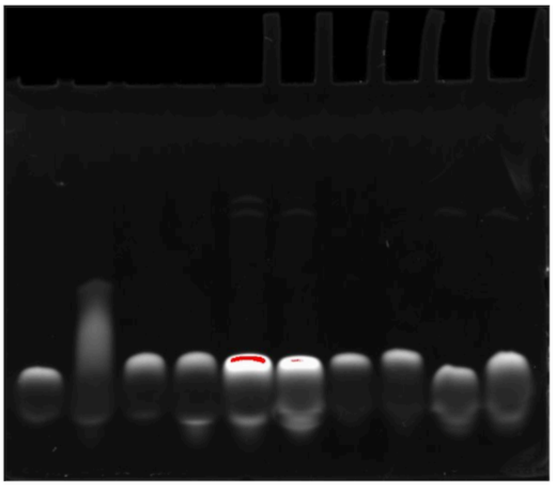

Supplementary Figure 4b

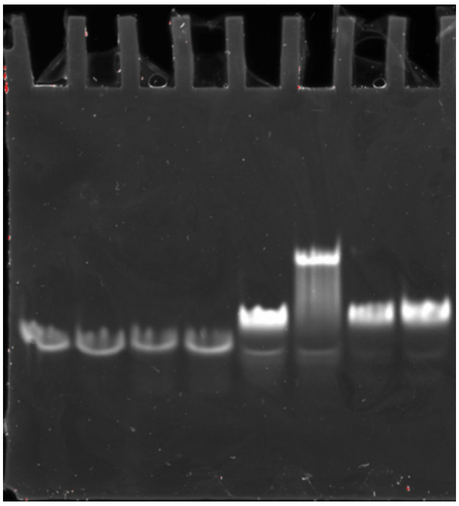

Supplementary Figure 5C (from L-R)

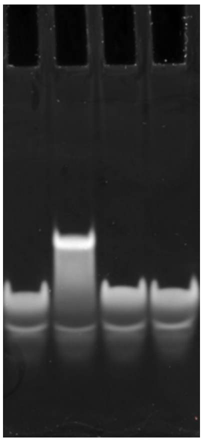

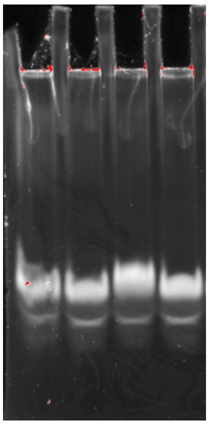

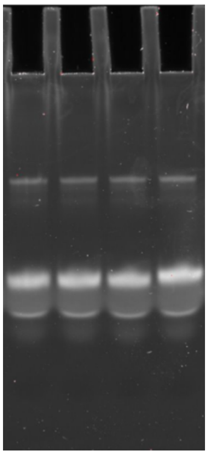

Supplementary Figure 5b (from L-R)

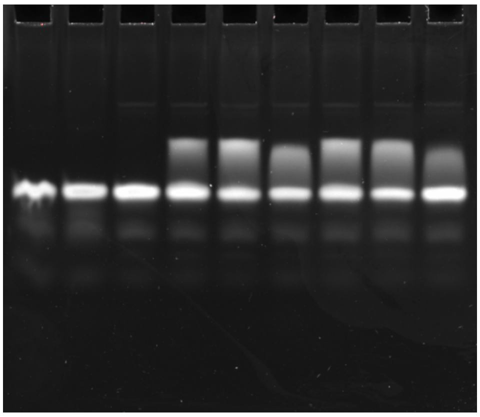

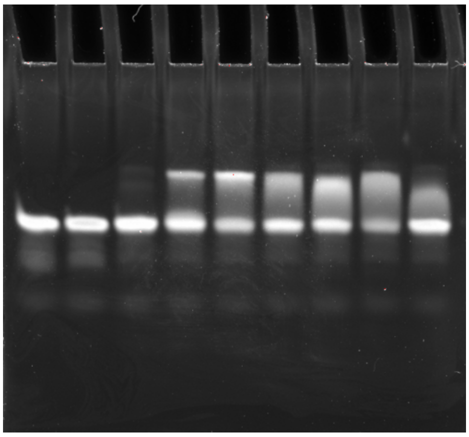

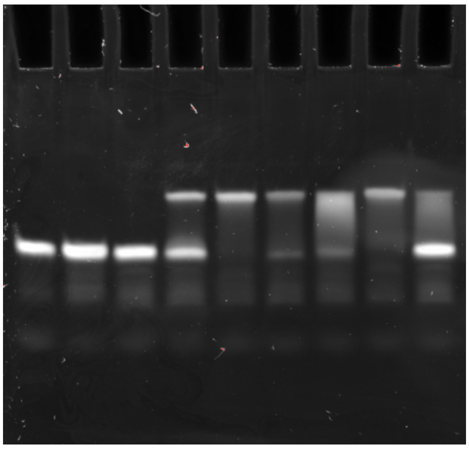

Supplementary Figure 5c

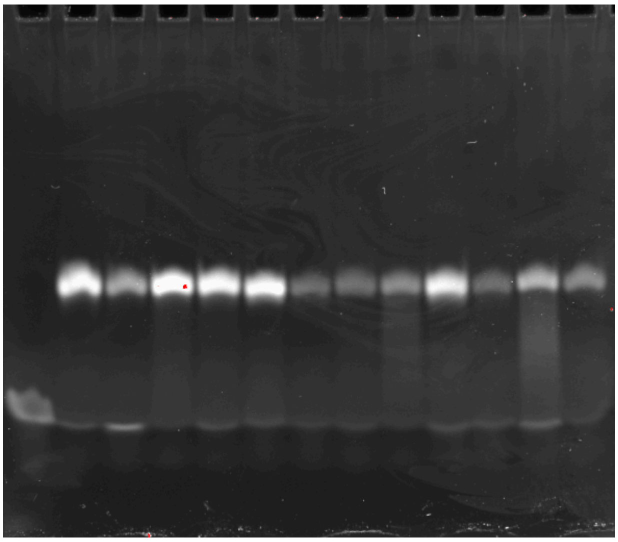

Supplementary Figure 8 (from L-R)

T1

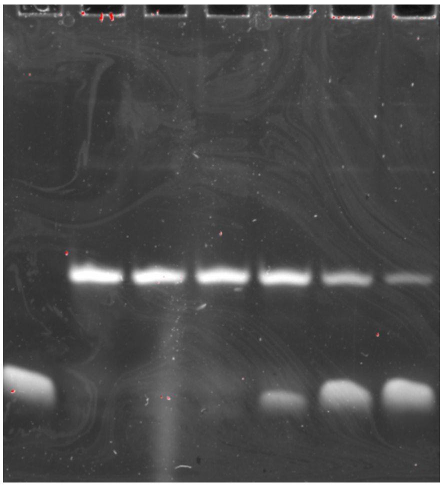

T2

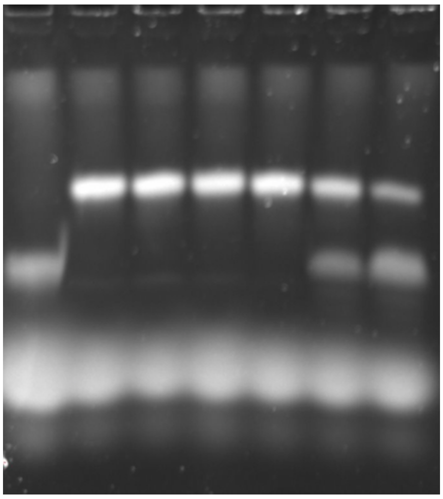

T3

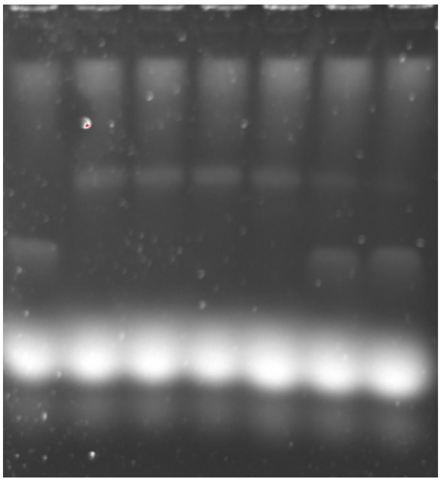

T4

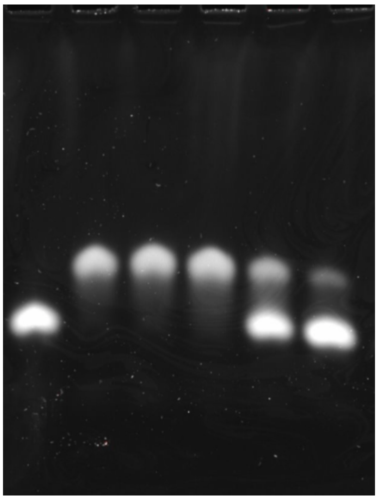

T5

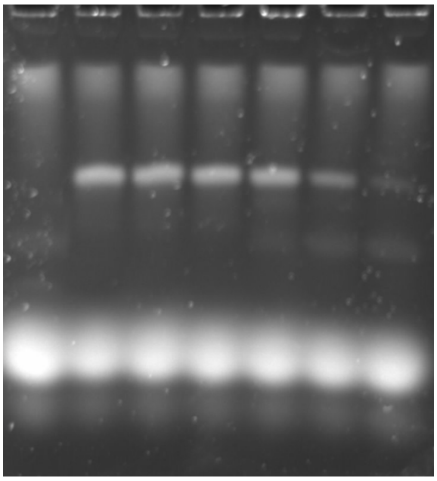

T6

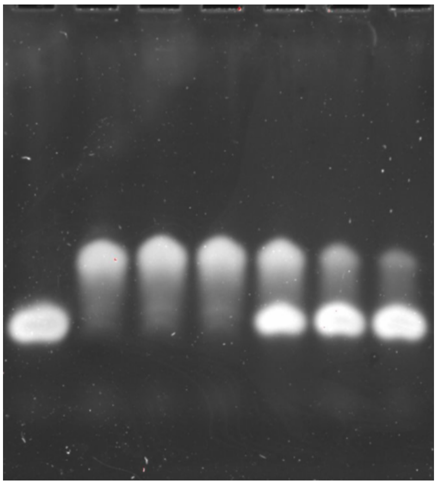

T7

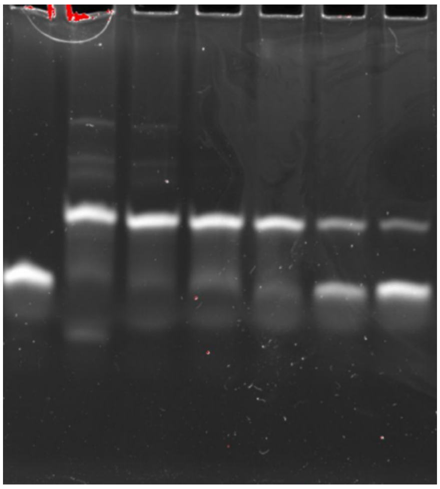

T8

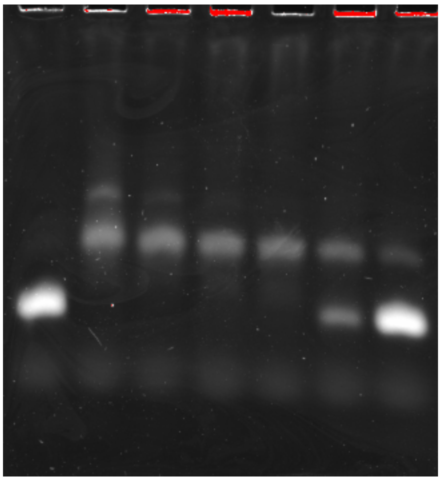

T9

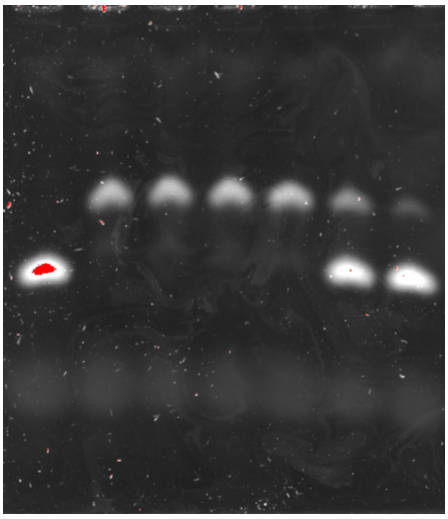

T10

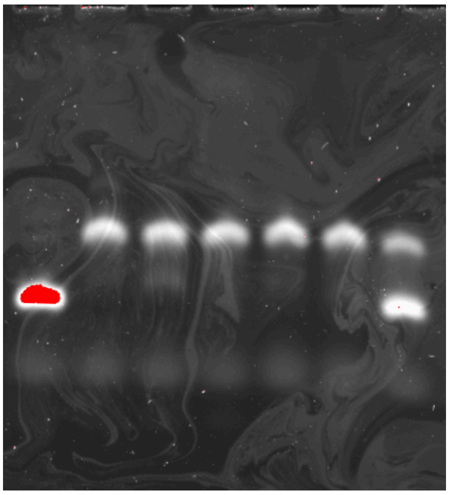

Supplementary Figure S13b

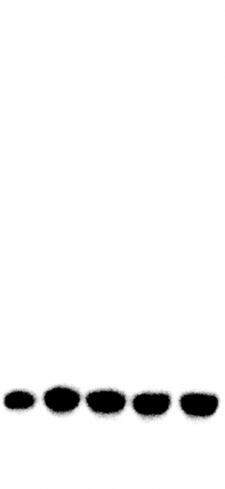

Supplementary Figure S13c

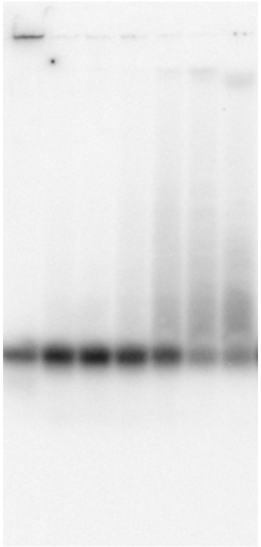

SUPPLEMENTARY REFERENCES

(1) Darby, R. A. J., Sollogoub, M., McKeen, C., Brown, L., Risitano, A., Brown, N., Barton, C., Brown, T., Fox, K. R. High Throughput Measurement of Duplex, Triplex and Quadruplex Melting Curves Using Molecular Beacons and a LightCycler. *Nucleic Acids Res.* **30**, e39 (2002).

(2) Xia, Y., Chu, W., Qi, Q., Xun, L. New Insights into the QuikChangeTM Process Guide the Use of Phusion DNA Polymerase for Site-Directed Mutagenesis. *Nucleic Acids Res.* **43**, e12–e12 (2015).

(3) Yang, Z., Sismour, A. M., Sheng, P., Puskar, N. L., Benner, S. A. Enzymatic Incorporation of a Third Nucleobase Pair. *Nucleic Acids Res.* **35**, 4238–4249 (2007).

(4) Singh, I., Laos, R., Hoshika, S., Benner, S. A. Georgiadis, M. M. Snapshots of an Evolved DNA Polymerase Pre- and Post-Incorporation of an Unnatural Nucleotide. *Nucleic Acids Res.* **46**, 7977-7988 (2018).

(5) Moody, E. R., Obexer, R., Nickl, F., Spiess, R., Lovelock, S. L. An Enzyme Cascade Enables Production of Therapeutic Oligonucleotides in a Single Operation. *Science* **380**, 1150–1154 (2023).

(6) Rusling, D. A. Triplex-Forming Properties and Enzymatic Incorporation of a Base-Modified Nucleotide Capable of Duplex DNA Recognition at Neutral pH. *Nucleic Acids Res.* **49**, 7256–7266 (2021).

(7) Gray, D. M., Hung, S. H., Johnson, K. H. Absorption and Circular Dichroism Spectroscopy of Nucleic Acid Duplexes and Triplexes. *Methods Enzym.* **246**, 19–34 (1995).
