# Supplementary material for: Recognition of non-standard base pairs by triplex-forming oligonucleotides containing an expanded genetic alphabet
Source: Nat Commun. 2026 Jun 12;17:7477. doi: 10.1038/s41467-026-74375-4 (PMC13407867; doi:10.1038/s41467-026-74375-4)
Supplement: Supplementary file 3 — Transparent Peer Review file [file 41467_2026_74375_MOESM3_ESM.pdf]

nature portfolio

Peer Review File

Recognition of Non-standard Base Pairs by Triplex-Forming Oligonucleotides Containing an Expanded Genetic Alphabet

Corresponding Author: Dr David Rusling

This file contains all reviewer reports in order by version, followed by all author rebuttals in order by version.

Version 0:

Reviewer comments:

Reviewer #1

(Remarks to the Author)
This is a nice study from Rusling's team which have expanded the triplex forming oligonucleotide recognition "catalogue" using nucleobases from an artificially expanded genetic information system. The team evaluated 120 base triplet combinations and have identified 12 new modular triplets that can be combined interchangeably to target duplex DNA. The system can be used for targeting normal B-form duplex, damaged and also synthetic base pairs. Importantly these have been identified to target at neutral pH. In addition to identifying the new combinations, they have showed some examples of their application herein. This work provides a dial-in platform for precision gene-targeting with applications for therapeutic design as well as other applications including "molecular sensing" and DNA-based nanotech. The work has many applications and I think has the potential to be used widely in the field. The work is sound and very well presented. The figures are clear. I think it is highly suitable for publishing in Nature Comms.
Comments:
1) Melting profiles in 3C. Normalisation of melt curves can give artifacts. Should have raw data plots in SI and comment on this. Also comment needed on the use of fluorescent dye and benefits/drawbacks of these as end-stacking can complicate melts with fluorescently labelled species etc. and why not use UV melts etc.
2) Some rogue uMs present
3) Table 1 needs errors included
4) Statistical significance needs to be discussed with changes observed
5) I wonder whether some sort of formatting is needed for the descriptions of the bases? It can get a bit difficult to read - can they be bold or italicised?
There is a lot of work here in this paper. It is easy for a reviewer to suggest more experiments, this is already substantive as it is.

Reviewer #2

(Remarks to the Author)
This manuscript presents a systematic investigation of the recognition of standard and non-standard DNA base pairs by triplex-forming oligonucleotides (TFOs) incorporating nucleobases from an artificially expanded genetic alphabet. The authors screen a large set of possible base triplets and evaluate their ability to form triplexes with duplexes containing canonical as well as modified base pairs.

Overall, the study is thorough and carefully executed. The experimental work appears solid, the data are well documented, and the figures and Supporting Information are extensive and clear. The systematic screening approach represents a significant effort and generates a valuable dataset for the field. The work convincingly demonstrates that the use of non-canonical nucleobases can broaden the scope of triplex recognition and potentially expand the toolbox for targeting duplex DNA sequences that are otherwise difficult to address with conventional TFOs.

Several results are particularly interesting. For example, the ability of certain triplets to recognize modified or non-canonical base pairs, including oxidized bases such as 8-oxoG, highlights potential applications in sensing or targeting damaged DNA. In addition, the study suggests that some of the triplets may allow discrimination between closely related base pairs, which could be useful for analytical or diagnostic applications.

The manuscript is generally well written and the experimental work appears comprehensive. The figures are clear and the Supporting Information provides useful supporting data.

At the same time, some aspects of the manuscript could be clarified or improved. In particular, although the dataset is extensive, the manuscript sometimes reads as a catalogue of observations rather than a distilled set of design principles for triplex recognition. A clearer summary of the key trends emerging from the screening would strengthen the conceptual impact of the work.

In addition, the authors describe their system as establishing an "orthogonal" triplet recognition code. However, the results indicate that both canonical and non-canonical nucleobases can interact with multiple partners, suggesting that the system is not strictly orthogonal in the usual sense of the term. Clarifying the intended meaning of this statement would improve the discussion.

Minor comments and suggestions

1. Ensure consistent notation for oxidized bases throughout the manuscript (e.g., 8oxoG vs 8oxG).

2. The term "triplet" is used both for a three-strand complex and for a base triad. In several places it may improve clarity to explicitly use "base triad".

3. Abstract (p.1 line 24): The statement that natural nucleobases require acidic conditions for stability applies specifically to parallel triplexes, which could be clarified.

4. Introduction (p.2 line 17): The reference supporting RNA-mediated triplex mechanisms is from 2015. More recent literature could better reflect the current state of the field.

5. p.3 line 5: The explanation of the notation X:R–Y appears prematurely, as it is not directly relevant to Figure 1 and may be better placed later in the text.

6. p.3 line 9: Instead of "purine-rich oligonucleotides," it may be clearer to explicitly refer to anti-parallel TFOs, which avoids requiring the reader to recall the purine/anti-parallel correspondence.

7. Figure 2:
The color gradients are somewhat distracting.
The notation for base pairs (R–Y) is not entirely consistent, especially where Y occasionally represents purines and where the order appears reversed in Figure S2.

8.Since Mg^{2+} concentration strongly influences triplex formation, the magnesium concentration should be clearly indicated in the figure captions of EMSA experiments.

9. Figure 3 and melting analysis:
Triplex melting curves often contain multiple transitions, and the manuscript should clarify how the melting temperatures were determined (e.g., midpoint vs first derivative).
A brief discussion of how overlapping duplex and triplex transitions influence interpretation would be helpful.

10.p.7 line 10: The text states that the unmodified CT TFO does not bind under neutral conditions, yet Figure 3 suggests that a single C-GC interaction can be tolerated, which could be clarified.

11.In several places the manuscript describes gels as demonstrating "stable triplex formation," although the gels sometimes show smearing or remaining duplex. The wording could be made slightly more cautious.

12. p.8 line 7: The text refers to experiments at higher magnesium concentrations, but the exact Mg^{2+} concentration is not clearly specified.

13. p.8 line 26:
• Clarify why ΨU is described as a C-nucleoside.
• The discussion of ΨC tautomerism may need clarification, since duplex formation likely fixes the tautomeric state.

14. p.9 line 23: Use consistent wording for figure references (e.g., "Figure 3c and S7").

15. p.10 line 13: The discussion of melting data would benefit from mentioning that triplex melting curves often reflect multiple transitions rather than a single event.

16. p.10 line 13: Likely T7–T10 rather than T6–T10.

17. p.11 line 11: T:B-Sc instead of T:B-S.

18. p.13 line 33: The heading appears grammatically incorrect and may require "in" instead of "is."

19. p.14 line 13: The stability comparison appears inaccurate: Kn:8oxoG-T appears slightly more stable than the other

triplets mentioned.

20. p.14 line 13: If the difference is ~8 °C, it would be more accurate to report that value rather than “~10 °C”.

21. Figure 5c: It is difficult to interpret the gel directly from the figure and the meaning of the 0–100 % scale is not immediately clear.

22. Figure 5d: Clarify why binding of the TFO produces such a large apparent migration shift in panel (i), lane 4.

23. p.16 line 32: Possibly B-Sc instead of B-S.

24. Figure 6 b(ii) and c: The concentrations of aTFO-T in each lane should be specified.

25. p.18 line 13 and p.19 line 8: The text refers to PAGE, while elsewhere the experiments are described as EMSA; terminology should be consistent.

26. Discussion: The claim that the authors have established an “orthogonal triplet recognition code” should be clarified, as the results indicate cross-recognition
between canonical and expanded bases.

27. Figure 7 and related discussion: The rationale for highlighting certain triplets (e.g., C:P-Z, C:ΨC-G) while omitting others (such as C:ΨU-A) could be explained.

28. Experimental section: The annealing protocol is somewhat unclear with respect to duplex vs triplex formation and could be described more explicitly.

29.Supporting Information:
Figure S4c: Clarify the meaning of **.
Figure S5 caption: likely c instead of d.
Figure S10 caption wording should be revised for clarity.
Consider using consistent axes across the melting plots for easier comparison.

Overall, the manuscript represents a substantial and valuable contribution to the field of nucleic acid chemistry. With clarification of several conceptual points and correction of the minor issues listed above, the work should be suitable for publication.

Reviewer #3

(Remarks to the Author)
The manuscript reports a series of synthetically modified DNA bases (AEGIS) which are able to recognise both normal and damaged sites in DNA duplexes when incorporated into triplex forming oligonucleotides. Recognition includes natural and damaged bases, with discrimination between these as a function of the TFO. The authors have extensively characterised this using a range of techniques including gels, fluorescence melting, UV/CD spectroscopy, ITC and PCR to validate the selectivity of TFO binding.
One of the main challenges limiting the application of TFOs is structural instability – the more stable parallel assembly requires low pH and the antiparallel assembly is far less stable. Given the sequence requirements of these assemblies, it is challenging to target the variety of natural DNA sequences that would be desirable within these restrictions.
Here the authors present an expanded series of bases which can be produced using standard phosphoramidite chemistry and are capable of recognising a variety of natural and modified sequences, to form stable assemblies which are pH independent.
The authors have comprehensively characterised the formation of the triplex, using mobility shift assays and fluorescence melting. The melting showed an increase in Tm across all modified sequences and the formation of the triplex is supported by the change in band positions of the triplex vs duplex for the assays. Thermal stability of the duplexes and triplex have been examined using fluorescence melting. Whilst I would have concern here that the interaction between SYBR green and the nucleic acid may affect the absolute Tm, given the SYBR green may stack onto the DNA and increase the stability of the assembly, the use of UV absorption and ITC to explore the Tm and thermodynamics of formation, mitigate this. The limitations of the approach, such as relatively low triplet stability of P-Z triplets, have also been highlighted with possible explanations (supported by the experimental evidence) given. Overall the experiments undertake are, in this reviewers opinion, fully comprehensive and have been undertaken correctly.
The outcomes of the work are significant, exciting and fully supported by the experimental evidence provided. Not only have the authors identified a set of modifications that allow them to target DNA duplexes with TFOs across a wide range of sequences but have also demonstrated both sequence-specific targeting and the ability to target an 8-oxoG-C base pair, extending the applications to DNA damage recognition. They have overcome the limitation of requiring low pH for stable triplex formation. The potential impact of this work is transformative within their own field and I strongly believe that the work be published in its current form.

Version 1:

Reviewer comments:

Reviewer #1

(Remarks to the Author)
I am happy with the responses made to the comments and supportive for publishing.

Reviewer #2

(Remarks to the Author)
The authors have carefully addressed my comments and suggestions, and I find their responses satisfactory. I therefore recommend that the manuscript be accepted without further changes.

Open Access This Peer Review File is licensed under a Creative Commons Attribution 4.0 International License, which permits use, sharing, adaptation, distribution and reproduction in any medium or format, as long as you give appropriate credit to the original author(s) and the source, provide a link to the Creative Commons license, and indicate if changes were made.
In cases where reviewers are anonymous, credit should be given to 'Anonymous Referee' and the source.
The images or other third party material in this Peer Review File are included in the article’s Creative Commons license, unless indicated otherwise in a credit line to the material. If material is not included in the article’s Creative Commons license and your intended use is not permitted by statutory regulation or exceeds the permitted use, you will need to obtain permission directly from the copyright holder.
To view a copy of this license, visit https://creativecommons.org/licenses/by/4.0/

We thank the Editor and Reviewers for their time and constructive comments on our manuscript. In response to their suggestions, we have corrected typographical errors, incorporated further clarifications, and added representative raw data to the SI. Our responses to comments are written in blue below and all amendments to the main text and/or SI are highlighted in red.

Reviewer #1:

This is a nice study from Rusling's team which have expanded the triplex forming oligonucleotide recognition "catalogue" using nucleobases from an artificially expanded genetic information system. The team evaluated 120 base triplet combinations and have identified 12 new modular triplets that can be combined interchangeably to target duplex DNA. The system can be used for targeting normal B-form duplex, damaged and also synthetic base pairs. Importantly these have been identified to target at neutral pH. In addition to identifying the new combinations, they have showed some examples of their application herein. This work provides a dial-in platform for precision gene-targeting with applications for therapeutic design as well as other applications including "molecular sensing" and DNA-based nanotech. The work has many applications, and I think has the potential to be used widely in the field. The work is sound and very well presented. The figures are clear. I think it is highly suitable for publishing in Nature Comms.

We thank the reviewer for their supportive comments and recommendation that our work is suitable for publication in *Nature Communications*.

Comments:

Melting profiles in 3C. Normalisation of melt curves can give artifacts. Should have raw data plots in SI and comment on this.

Melting profiles for each complex in Figure 3C were normalised to fluorescence at 40 °C ($F_{norm}(T) = F(T)/F_{40\ °C}$) to account for instrument variability (dye loading, detector gain, etc), while preserving the shape of the transition. Importantly, T_m values were extracted from the first derivatives of the raw (non-normalised) data using instrument software. We have now included representative raw melting curves in the SI (Figure S7b) and updated the legend accordingly.

Also comment needed on the use of fluorescent dye and benefits/drawbacks of these as end-stacking can complicate melts with fluorescently labelled species etc. and why not use UV melts etc.

Fluorescence melting using SYBR Green I on a qPCR platform is a well-established, high throughput approach for ***comparative*** stability screening of nucleic acid complexes. Compared with conventional UV melting, this approach requires substantially lower sample volumes (20 µL *vs* 100 µL) and oligonucleotide concentrations (1 µM *vs* 5 µM), while enabling high-throughput analysis (up to 32 samples in parallel *vs* ~5) (added Ref. 41). Previous studies have shown that SYBR Green I also fluoresces upon binding to triplex DNA (added Ref. 42), and we have exploited this property to characterise triplex formation in earlier work (Ref. 19). Due to its DNA-binding

mode, SYBR Green will lead to a minor stabilisation of both duplex and triplex complexes that will affect the absolute T_m. However, and as noted by Reviewer 3, key findings were corroborated in the absence of SYBR green I for many of the complexes by EMSA, UV, CD, and ITC analysis.

We have made the following text amendments (and added references) to highlight the applicability of this technique, while also noting associated caveats:

"To quantify the thermal stability of the triads identified by EMSA analysis, we then performed fluorescence melting using SYBR Green I and a qPCR machine (**Figure 3c** and **S7**).[41] SYBR green I fluoresces upon binding to duplex and triplex DNA,[42] and the melting of the complexes results in a decrease in fluorescence intensity at 522 nm.[19] Duplexes were first annealed at pH 7.0 in sodium cacodylate buffer containing 10 mM magnesium and subsequently incubated with the appropriate TFO before melting analysis. Triplex formation was identified by an upward shift in the melting profile relative to that of the corresponding duplex-only control. In some cases, we observed a minor drop in fluorescence at lower temperatures that can be attributed to melting of unbound duplexes in the samples. Melting temperatures (T_m) were therefore determined from the first derivatives of the highest melting transition and used to evaluate the relative stability of each triad. Due to its DNA binding mode, SYBR green I binding will lead to a slight stabilization of both the duplex and triplex complexes, and the T_m values determined will not equate to absolute T_m values obtained in the absence of the dye."

Some rogue uMs present.

Corrected, many thanks.

Table 1 needs errors included. Statistical significance needs to be discussed with changes observed.

This is an excellent point, but we are not sure if the reviewer is referring to T_m measurements in Figure 3, or Table 1 itself. In both cases we had reported mean T_m values but not included standard errors. Consequently, we have now added standard errors for both sets of data and updated both the Figure and Table.

Analysis of the errors highlights the reproducibility of the fluorescence melting assay, with T_m values typically varying by less than 1 °C between experiments. Differences in T_m that exceed the associated standard error (typically <1 °C) were considered significant in the context of this study, particularly given the large dynamic range observed (>10 °C across base triads). However, we included a cautionary note regarding assessing the stabilization afforded by nucleobase modifications based on thermal melting alone:

"However, previous work demonstrated that triplex stability is influenced by the stability of the underlying duplex and, consequently, thermal melting can underestimate stabilization afforded by nucleobase modifications.[43]"

We believe that including error analysis strengthens the robustness of the SYBR Green melting assay and provides clear assessment of reproducibility across replicates, thanks.

I wonder whether some sort of formatting is needed for the descriptions of the bases? It can get a bit difficult to read - can they be bold or italicised?

Good suggestion and we have now indicated all third strand bases in bold (**T**, **C**, **Z**, **V**, **S^c**, **K^n**) throughout the main text and SI. We believe this improves the clarity of the manuscript, but we leave it to the Editorial Team to decide whether this format is appropriate (note: these are not shown in red in the amended main text and SI and are just shown in bold to highlight the formatting change).

There is a lot of work here in this paper. It is easy for a reviewer to suggest more experiments; this is already substantive as it is.

Again, many thanks for the time taken to read our manuscript and provide constructive comments.

Reviewer #2

This manuscript presents a systematic investigation of the recognition of standard and non-standard DNA base pairs by triplex-forming oligonucleotides (TFOs) incorporating nucleobases from an artificially expanded genetic alphabet. The authors screen a large set of possible base triplets and evaluate their ability to form triplexes with duplexes containing canonical as well as modified base pairs. Overall, the study is thorough and carefully executed. The experimental work appears solid, the data are well documented, and the figures and Supporting Information are extensive and clear. The systematic screening approach represents a significant effort and generates a valuable dataset for the field. The work convincingly demonstrates that the use of non-canonical nucleobases can broaden the scope of triplex recognition and potentially expand the toolbox for targeting duplex DNA sequences that are otherwise difficult to address with conventional TFOs.

Several results are particularly interesting. For example, the ability of certain triplets to recognize modified or non-canonical base pairs, including oxidized bases such as 8-oxoG, highlights potential applications in sensing or targeting damaged DNA. In addition, the study suggests that some of the triplets may allow discrimination between closely related base pairs, which could be useful for analytical or diagnostic applications.

The manuscript is generally well written, and the experimental work appears comprehensive. The figures are clear and the Supporting Information provides useful supporting data.

We thank the reviewer for their favourable comments and taking the time to extensively proofread both the main text and SI.

At the same time, some aspects of the manuscript could be clarified or improved. In particular, although the dataset is extensive, the manuscript sometimes reads as a catalogue of observations rather than a distilled set of design principles for triplex recognition. A clearer summary of the key trends emerging from the screening would strengthen the conceptual impact of the work.

We agree that, due to the nature of these screening experiments, the results section of the manuscript reads as a catalogue of observations. To address this, we have amended parts of the main text and much of the discussion.

In addition, the authors describe their system as establishing an "orthogonal" triplet recognition code. However, the results indicate that both canonical and non-canonical nucleobases can interact with multiple partners, suggesting that the system is not strictly orthogonal in the usual sense of the term. Clarifying the intended meaning of this statement would improve the discussion.

This is an important point, and we agree that the system is not strictly orthogonal in the sense of one-to-one base triad recognition. We have therefore clarified the terminology throughout the manuscript to emphasise that the expanded nucleobases enable a programmable and partially orthogonal recognition code, in which the third strand nucleobases exhibit preferential—but not exclusive—interactions. This reflects the inherent flexibility of Hoogsteen recognition and is now mentioned explicitly in the revised Discussion section.

Minor comments and suggestions:

Ensure consistent notation for oxidized bases throughout the manuscript (e.g., 8oxoG vs 8oxG).

Corrected, many thanks.

The term "triplet" is used both for a three-strand complex and for a base triad. In several places it may improve clarity to explicitly use "base triad".

We have now replaced the word "triplet" with "triad" throughout the main text and SI.

Abstract (p.1 line 24): The statement that natural nucleobases require acidic conditions for stability applies specifically to parallel triplexes, which could be clarified.

Clarified, many thanks.

Introduction (p.2 line 17): The reference supporting RNA-mediated triplex mechanisms is from 2015. More recent literature could better reflect the current state of the field.

We have now replaced the dated citation with an excellent study that points to a general triplex-mediated gene regulatory mechanism:

11. Genna, V. *et al.* Systematic study of hybrid triplex topology and stability suggests a general triplex-mediated regulatory mechanism. *Nucleic Acids Res.* **53**, gkaf170 (2025).

p.3 line 5: The explanation of the notation X:R–Y appears prematurely, as it is not directly relevant to Figure 1 and may be better placed later in the text.

We appreciate the reviewer's suggestion. However, we have retained the original placement, as it introduces the notation immediately following statements describing specific parallel and antiparallel base triplets. Given that some research groups represent triads in the reverse Y–R:X format, we felt it important to define our notation clearly from the outset of the manuscript.

p.3 line 9: Instead of "purine-rich oligonucleotides," it may be clearer to explicitly refer to anti-parallel TFOs, which avoids requiring the reader to recall the purine/anti-parallel correspondence.

We have now amended to "Antiparallel oligonucleotides rich in purines…"

Figure 2: The color gradients are somewhat distracting.

Due to the number of datasets presented and page and figure constraints, colour gradients were used to aid visualisation of the data. Since many of these profiles are overlapping, we have included separated melting profiles in the SI (Figure S7).

The notation for base pairs (R–Y) is not entirely consistent, especially where Y occasionally represents purines and where the order appears reversed in Figure S2.

We opted to use R-Y to indicate any given base pair and not specifically a purine-pyrimidine base pair. We chose R to reflect the base in the purine-rich (R) strand, and Y to reflect the base in the pyrimidine-rich (Y) strand. We have now clarified this in the main text as follows:

By systematically positioning each nucleobase opposite each base pair in turn, we evaluated 120 distinct **X**:R-Y triad combinations, where R and Y indicate bases positioned in the purine-rich and pyrimidine-rich strand of the duplex, respectively.

Since Mg^{2+} concentration strongly influences triplex formation, the magnesium concentration should be clearly indicated in the figure captions of EMSA experiments.

Apologies, we have now added specific magnesium concentrations in the main text, SI, and all figure legends.

Figure 3 and melting analysis: Triplex melting curves often contain multiple transitions, and the manuscript should clarify how the melting temperatures were determined (e.g., midpoint vs first derivative).

We have added the following to the main text:

In some cases, we observed a minor drop in fluorescence at lower temperatures that can be attributed to the melting of unbound duplexes in the samples. Melting temperatures (T_m) were therefore determined from the first derivatives of the highest melting transition and used to evaluate the relative stability of each triad.

A brief discussion of how overlapping duplex and triplex transitions influence interpretation would be helpful.

Following on from the previous comment, we have made the following text amendments (and added references) to highlight the applicability of using the fluorescence melting technique to bolster EMSA data, whilst also noting the associated caveats:

"To quantify the thermal stability of the triads identified by EMSA analysis, we then performed fluorescence melting using SYBR Green I and a qPCR machine (**Figure 3c** and **S7**).[41] SYBR green I fluoresces upon binding to duplex and triplex DNA,[42] and the melting of the complexes results in a decrease in fluorescence intensity at 522 nm.[19] Duplexes were first annealed at pH 7.0 in sodium cacodylate buffer containing 10 mM magnesium and subsequently incubated with the appropriate TFO before melting analysis. Triplex formation was identified by an upward shift in the melting profile relative to that of the corresponding duplex-only control. In some cases, we observed a minor drop in fluorescence at lower temperatures that can be attributed to the melting of unbound duplexes in the samples. Melting temperatures (T_m) were therefore determined from the first derivatives of the highest melting transition and used to evaluate the relative stability of each triad. Due to its DNA binding mode, it should be noted that SYBR green I will lead to a slight stabilization of both the duplex and triplex complexes, and the T_m values determined will not equate to the absolute T_m values determined in the absence of the dye."

p.7 line 10: The text states that the unmodified CT TFO does not bind under neutral conditions, yet Figure 3 suggests that a single C-GC interaction can be tolerated, which could be clarified.

This is an insightful observation, and we have added the following to the main text:

Notably, the C:G-C triplet was tolerated in this stable sequence context, despite experiments being conducted at neutral pH.

In several places the manuscript describes gels as demonstrating "stable triplex formation," although the gels sometimes show smearing or remaining duplex. The wording could be made slightly more cautious.

We have moderated the wording throughout to avoid overinterpretation of EMSA data, replacing "stable triplex formation" with more precise descriptions where appropriate. For example:

However, some smearing and remaining duplex was observed for the triplex containing the **S**^{c}:8oxA-**T** triad, indicating it was of lower stability compared to the other triads.

p.8 line 7: The text refers to experiments at higher magnesium concentrations, but the exact Mg^{2+} concentration is not clearly specified.

We have corrected all magnesium concentrations in the main text, SI and all figure legends.

13. p.8 line 26: Clarify why ΨU is described as a C-nucleoside.

We believe this terminology is correct, as ΨU is a non-canonical C-glycoside isomer of uridine.

The discussion of ΨC tautomerism may need clarification, since duplex formation likely fixes the tautomeric state.

Excellent point and we have amended the text as follows:

…although the recognition of ΨC will depend on its tautomeric equilibrium which might be fixed upon duplex formation.[40]

14. p.9 line 23: Use consistent wording for figure references (e.g., "Figure 3c and S7").

We believe we have used consistent wording for figure references, for example Figure 3c refers to a figure in the main text, and Figure S7 in the supplementary. Perhaps this is not appropriate, and we will leave it to the Editorial Team to advise.

15. p.10 line 13: The discussion of melting data would benefit from mentioning that triplex melting curves often reflect multiple transitions rather than a single event.

Please see comment above.

16. p.10 line 13: Likely T7–T10 rather than T6–T10.

Well spotted – corrected.

17. p.11 line 11: T:B-Sc instead of T:B-S.

Corrected, many thanks.

p.13 line 33: The heading appears grammatically incorrect and may require "in" instead of "is."

Corrected, appreciated.

19. p.14 line 13: The stability comparison appears inaccurate: Kn:8oxoG-T appears slightly more stable than the other triplets mentioned.

Corrected, many thanks.

p.14 line 13: If the difference is ~8 °C, it would be more accurate to report that value rather than "~10 °C".

Corrected, thanks.

Figure 5c: It is difficult to interpret the gel directly from the figure and the meaning of the 0–100 % scale is not immediately clear.

We have corrected the figure legend to make this clearer.

Figure 5d: Clarify why binding of the TFO produces such a large apparent migration shift in panel (i), lane 4.

The gel in figure 5d(i) is an agarose gel assessing the reaction products of the SDM assay, while the gel in Figure 5d(ii) assessed the binding of the TFO to these SDM products. We think the confusion lies in the use of a black asterisk next to the gel in question, which elsewhere we have used to indicate the formation of a triplex. We see how this might be confusing and have now removed the black asterisk from the gel and amended the figure legend accordingly.

p.16 line 32: Possibly B-Sc instead of B-S.

Corrected, well spotted.

Figure 6 b(ii) and c: The concentrations of aTFO-T in each lane should be specified.

We apologise for missing this information and have updated the figure legend as follows:

.. the assembled TFO at a concentration of approximately 250, 100, 25, 10, 25, 1 nM (lanes 3-8, respectively) with the synthetic duplex sequence containing B-S^c and P-Z at 1 μM (ii). (c) Combining the products of each reaction from (a) and (b) allowed recognition of an enzymatically assembled AEGIS duplex by an enzymatically generated TFO containing AEGIS nucleobases. The assembled TFO was at a concentration of approximately 250, 100, 25, 10, 25, 1 nM (lanes 3-8, respectively) and the assembled duplex containing B-S^c and P-Z at approximately 0.25 μM.

p.18 line 13 and p.19 line 8: The text refers to PAGE, while elsewhere the experiments are described as EMSA; terminology should be consistent.

Corrected, many thanks.

Discussion: The claim that the authors have established an "orthogonal triplet recognition code" should be clarified, as the results indicate cross-recognition between canonical and expanded bases.

Please see above comment and we have amended the Discussion.

Figure 7 and related discussion: The rationale for highlighting certain triplets (e.g., C:P-Z, C:ΨC-G) while omitting others (such as C:ΨU-A) could be explained.

We have gone some way to address this point in detail and have now included a discussion of all base triads that were shown to form from screening. Putative structures of the triads that were not taken forward during the study, on account of their lower stability and/or formation of less conventional backbone geometries, are now shown in Figure S14.

Experimental section: The annealing protocol is somewhat unclear with respect to duplex vs triplex formation and could be described more explicitly.

We have amended the text to the following:

"Duplex strands were annealed by heating the strands at 95 °C for 5 minutes and slowly cooling the samples to room temperature over 2 hours. The TFOs were then added to the annealed complexes and left to equilibrate for greater than 16 hours at 4 °C."

Supporting Information: Figure S4c: Clarify the meaning of **.

The double "**" is used to indicate minor species that correspond to unintended interactions between G-rich and/or C-rich duplex strands in the Duplex 3G-C samples that a long run of Gs or Cs. We have clarified this in the figure legend:

The "*" is used to indicate species that likely correspond to the formation of a triplex, while "**" is used to indicate minor bands present in the duplex-only and triplex samples that likely correspond to unintended interactions between the G-rich or C-Rich duplex strands (Duplex 3G-C).

Figure S5 caption: likely c instead of d.

Corrected, many thanks.

Figure S10 caption wording should be revised for clarity.

We have revised the caption as follows:

Figure S10 – Modular recognition of different base pair combinations (UV melting). Triplex motifs analysed are indicated above each melting profile and differ in the type and number of modified base triads (T1–T10). Oligonucleotides were annealed in pH 7.0 sodium cacodylate buffer containing 10 mM Mg^{2+}, with final duplex and TFO concentrations of 5 and 10 μM, respectively. Samples were heated at 0.2 °C min^{-1} and absorbance monitored using a Shimadzu UV-3600 UV–Vis–NIR spectrophotometer. Melting temperatures (T_m) correspond to the midpoint of the thermal transition. Six triplexes exhibited increased T_m values relative to the corresponding duplex-only controls, indicating enhanced stability. Thermal stability was dependent on the identity of the base triad. Triplexes containing Y5-derivatives and AEGIS base pairs displayed melting profiles comparable to the underlying duplex, consistent with coincident duplex and triplex melting transitions.

Overall, the manuscript represents a substantial and valuable contribution to the field of nucleic acid chemistry. With clarification of several conceptual points and correction of the minor issues listed above, the work should be suitable for publication.

We again thank the reviewer for their careful and constructive evaluation. The suggested clarifications and corrections have improved the clarity and conceptual framing of the manuscript substantially.

Reviewer #3

The manuscript reports a series of synthetically modified DNA bases (AEGIS) which are able to recognise both normal and damaged sites in DNA duplexes when incorporated into triplex-forming oligonucleotides. Recognition includes natural and damaged bases, with discrimination between these as a function of the TFO. The authors have extensively characterised this using a range of techniques including gels, fluorescence melting, UV/CD spectroscopy, ITC and PCR to validate the selectivity of TFO binding.

One of the main challenges limiting the application of TFOs is structural instability – the more stable parallel assembly requires low pH, and the antiparallel assembly is far less stable. Given the sequence requirements of these assemblies, it is challenging to target the variety of natural DNA sequences that would be desirable within these restrictions.

Here the authors present an expanded series of bases which can be produced using standard phosphoramidite chemistry and are capable of recognising a variety of natural and modified sequences, to form stable assemblies which are pH independent.

The authors have comprehensively characterised the formation of the triplex, using mobility shift assays and fluorescence melting. The melting showed an increase in Tm across all modified

sequences, and the formation of the triplex is supported by the change in band positions of the triplex vs duplex for the assays. Thermal stability of the duplexes and triplex have been examined using fluorescence melting. Whilst I would have concern here that the interaction between SYBR green and the nucleic acid may affect the absolute Tm, given the SYBR green may stack onto the DNA and increase the stability of the assembly, the use of UV absorption and ITC to explore the Tm and thermodynamics of formation, mitigate this. The limitations of the approach, such as relatively low triplet stability of P-Z triplets, have also been highlighted with possible explanations (supported by the experimental evidence) given. Overall, the experiments undertake are, in this reviewer's opinion, fully comprehensive and have been undertaken correctly.

The outcomes of the work are significant, exciting and fully supported by the experimental evidence provided. Not only have the authors identified a set of modifications that allow them to target DNA duplexes with TFOs across a wide range of sequences but have also demonstrated both sequence-specific targeting and the ability to target an 8-oxoG-C base pair, extending the applications to DNA damage recognition. They have overcome the limitation of requiring low pH for stable triplex formation. The potential impact of this work is transformative within their own field, and I strongly believe that the work be published in its current form.

We thank the reviewer for their careful evaluation and strong support for publication of our manuscript. We are particularly encouraged that the reviewer recognises both the scope and rigor of the experimental approach, as well as the significance of expanding triplex recognition to include both canonical and damaged DNA base pairs under physiologically relevant (neutral pH) conditions.

We appreciate the reviewer's comments regarding the potential influence of SYBR Green on absolute T_m values. As noted, and now further clarified in the revised manuscript, key findings from fluorescence melting were corroborated using orthogonal techniques including UV melting, CD spectroscopy, EMSA and ITC, ensuring that the observed trends in triplex stability and selectivity are robust and not artefacts of dye binding. Inclusion of T_m error analysis also strengthens the conclusions of the manuscript (*e.g.,* Figure 3C).

We are also grateful for the recognition of the broader implications of this work, particularly in overcoming long-standing limitations associated with triplex stability and sequence scope. We believe that the ability to target both canonical and chemically modified DNA, including oxidatively damaged bases such as 8-oxoG, highlights the potential of this platform for applications in gene targeting, molecular sensing, and DNA nanotechnology.
